# Environmental factors contributing to the convergence of bacterial community structure during indigo reduction

**DOI:** 10.3389/fmicb.2023.1097595

**Published:** 2023-02-09

**Authors:** Nowshin Farjana, Zhihao Tu, Hiromitsu Furukawa, Isao Yumoto

**Affiliations:** ^1^Bioproduction Research Institute, National Institute of Advanced Industrial Science and Technology (AIST), Sapporo, Japan; ^2^Laboratory of Environmental Microbiology, Graduate School of Agriculture, Hokkaido University, Sapporo, Japan; ^3^Sensing System Research Center, National Institute of Advanced Industrial Science and Technology (AIST), Sapporo, Japan

**Keywords:** indigo reduction ecosystem, alkaline environment, alkaliphilic bacteria, alkaline treatment, convergence of microbiota

## Abstract

Indigo is solubilized through the reducing action of the microbiota that occurs during alkaline fermentation of composted leaves of *Polygonum tinctorium* L. (*sukumo*). However, the environmental effects on the microbiota during this treatment, as well as the mechanisms underlying the microbial succession toward stable state remain unknown. In this study, physicochemical analyses and Illumina metagenomic sequencing was used to determine the impact pretreatment conditions on the subsequent initiation of bacterial community transition and their convergence, dyeing capacity and the environmental factors critical for indigo reducing state during aging of *sukumo*. The initial pretreatment conditions analyzed included 60°C tap water (heat treatment: batch 1), 25°C tap water (control; batch 2), 25°C wood ash extract (high pH; batch 3) and hot wood ash extract (heat and high pH; batch 4), coupled with successive addition of wheat bran from days 5 to 194. High pH had larger impact than heat treatment on the microbiota, producing more rapid transitional changes from days 1 to 2. Although the initial bacterial community composition and dyeing intensity differed during days 2–5, the microbiota appropriately converged to facilitate indigo reduction from day 7 in all the batches, with *Alkaliphilus oremalandii*, *Amphibacillus*, *Alkalicella caledoniensis*, *Atopostipes suicloalis* and *Tissierellaceae* core taxa contributing to the improvement of when the dyeing intensity. This convergence is attributed to the continuous maintenance of high pH (day 1 ~) and low redox potential (day 2~), along with the introduction of wheat bran at day 5 (day 5~). PICRUSt2 predictive function profiling revealed the enrichment of phosphotransferease system (PTS) and starch and sucrose metabolism subpathways key toward indigo reduction. Seven NAD(P)-dependent oxidoreductases KEGG orthologs correlating to the dyeing intensity was also identified, with *Alkalihalobacillus macyae*, *Alkalicella caledoniensis*, and *Atopostipes suicloalis* contributing significantly toward the initiation of indigo reduction in batch 3. During the ripening period, the staining intensity was maintained by continuous addition of wheat bran and the successive emergence of indigo-reducing bacteria that also contributed to material circulation in the system. The above results provide insight into the interaction of microbial system and environmental factors in *sukumo* fermentation.

## Introduction

Indigo fermentation is facilitated by the reducing function of a microbial community based on the natural fermentation. There are two sources of natural indigo dye, namely precipitated indigo (extracted indigo) and composted plants that contain indigo. Precipitated indigo has been used as a source of indigo dye in India, China, Southeast Asia, and Japan (Okinawa Prefecture) (Toyama et al., 1978; Li et al., 2019, 2022; Lopes et al., 2021). Since the microorganisms in the seeds are not associated with the precipitated indigo for fermentation, spontaneous initiation of indigo reduction can be time consuming. Therefore, for earlier initiation of indigo reduction, it is necessary to add a seed culture (e.g., previous fermentation fluid or materials extracted from plants) that contains substrates for microorganisms, chemicals that act as electron mediators or oxygen eliminators, and other materials that create suitable conditions that facilitate indigo reduction (Lopes et al., 2021). Whether indigo reduction depends on the fermentation batch remains uncertain since it is a natural fermentation. The most popular procedure for reducing indigo is *via* sodium dithionite (Etters, 1989), which is not environmentally friendly. Therefore, it is desirable to establish a highly reliable, and environmentally friendly fermentation method based on the fundamentals of microflora formation to induce indigo reduction especially when using extracted indigo.

Procedures involving the composting of plants that contain indigo have been previously performed in Europe (Clark et al., 1993; Padden et al., 2000; Osimani et al., 2012; Hartl et al., 2015; Milanović et al., 2017). The composting process increases indigo concentration compared with the concentrations of the input plant and is more suitable for preservation and transportations. In this case, seed cultures for the indigo-reducing fermentation are derived from composting materials. The seed culture would comprise post-composted plant debris, microbial metabolites, and dead microorganismal cells, which serve as the microbial substrates in the fermented liquor. The composted indigo plant *Polygonum tinctorium* L. (*sukumo*) is a traditional indigo-dyeing material in Japan (Aino et al., 2018; Nakagawa et al., 2021). Composting of indigo plant is performed on an indoor earthen floor and is initiated by adding water to a pile of the dried indigo plant leaves. This process takes approximately 100 days, wherein the appropriate turnover frequency and water addition of the pile are maintained to enable continuous aerobic microbial digestion; this process requires a trained craftsperson. The fermentation temperature (center temperature) of the piled indigo leaves is approximately 70°C. The *sukumo* production process produces indigo (indigotin) from the precursor indican *via* indoxyl.

The period from the fermentation preparation to indigo reduction initiation differs depending on the procedure and quality of raw materials. Transitional changes in the redox state of indigo may be related to changes in the microbiota, which is in turn, associated with the indigo reduction initiation, as previously have suggested using clone library analysis (Aino et al., 2010) and next-generation sequencing (Tu et al., 2019a,b, 2021; Lopes et al., 2021, 2022; Nakagawa et al., 2022). On days 0–1, oxygen metabolizable *Bacillaceae* (e.g., *Sutcliffiella cohnii*) and *Actinobacteria* (e.g., *Nocardiopsis ganjiahuensis*) appear, following which their numbers significantly decrease during days 2–10 as the redox potential (ORP) decreases due to their consumption of oxygen. From days 2 to 5, the more anaerobic conditions become favorable for *Bacillaceae* [e.g., *Amphibacillus indicireducens* (type strain: C40^T^; Hirota et al., 2013a), *Amphibacillus iburiensis* (type strain: N314^T^; Hirota et al., 2013b) and *Alkalihalobacillus* spp. (formerly *Bacillus* spp.; Nishita et al., 2017)], obligate anaerobes [e.g., *Alkalicella* spp. (formerly *Proteinivoraceae* or *Anaerobranca*) and *Alkaliphilus* spp.], and *Lactobacillales* [e.g., *Alkalibacterium psychrotolerans* (type strain: IDR2-2^T^; Yumoto et al., 2004), *Alkalibacterium iburiense* (type strain: M3^T^: Nakajima et al., 2005), and *Alkalibacterium indicireducens* (type strain: A11^T^: Yumoto et al., 2008)], which begin to appear. Concomitant with these transitional changes in the microbiota, indigo reduction is initiated on days 4–7. The changing velocity of the microbiota is more rapid during the early period of fermentation then the later periods, e.g., later than day 20 (Okamoto et al., 2017; Tu et al., 2021). At a later phase (e.g., later than day 100), wheat bran decomposers [e.g., *Amphibacillus* spp. and *Polygonibacillus* spp. (Hirota et al., 2016, 2020)] and/or obligate anaerobes (e.g., *Proteinivoraceae* and/or *Anerobacillus*) become the dominant members in the fermentation fluids (Okamoto et al., 2017; Tu et al., 2021).

Appropriate selection for the initial microbial community is essential for the earlier initiation of indigo reduction since the microbiota of *sukumo* are different from that of the fermentation fluid (Tu et al., 2019a, 2021). The initial preparation procedure differs based on the craft center. Therefore, a standard procedure for the preparation of indigo fermentation remains lacking. For example, one craftsperson may treat *sukumo* with room temperature wood ash extract, while another may use hot water at the beginning. However, treating *sukumo* with hot wood ash extract (60°C–70°C, pH > 10.5) has been commonly conducted. Although the initial condition for fermentation is important for the early initiation of indigo reduction, the influence of environmental factors on the initial treatment for the transitional changes of microbial community in the fermentation fluid remains unclear.

Indigo fermentation employing *sukumo* is performed by natural fermentation. Therefore, it is difficult to prepare the same fermentation fluid in the microbiota due to subtle differences in pH and temperature producing different microbial communities. However, fermentation fluids with different microbiota proceed to the indigo-reducing state using the appropriate maintenance procedures (e.g., maintaining pH and gently stirring the fluid once a day). In addition, indigo fermentation includes two different phases regardless of their preparations (Tu et al., 2021). Appropriately prepared and maintained indigo fermentation fluid exhibits exquisite robustness that retains the indigo dyeing function for more than 1 year, even with continuous fabric dyeing in an open-air environment. Although the continuous presence of indigo reducing bacteria has been previously reported (Okamoto et al., 2017; Tu et al., 2021), the relationship between the environmental factors, dyeing intensity, and core indigo reducing bacteria base on the aging state remains to be determined.

Therefore, in this study, we determined the selective factors for the microbiota involved in the raw material among 4 different methods. Herein, we show that the microbiota exhibited independent feature based on each pretreatment. In addition, the intensity of the transitional change in the microbiota influenced by the pH maintenance is larger based on pretreatment with high pH and low redox potential, while introduction of wheat bran converges the microbiota to facilitate indigo reduction. Furthermore, we analyzed the relationship between indigo reduction and existing taxa in the fluid and their predicted functions. Analyzing the relationship between the environmental factors and the microbiota’s transitions under extreme conditions provides insights into the complex microbial for better management.

## Materials and methods

### Preparation of indigo fermentation fluid

Four different small-scale batches were prepared to evaluate the effect of the initial treatment of *sukumo* (produced by A.S. Tokushima, Shikoku, Japan) on indigo reduction initiation. *Sukumo* (76 g/batch) was treated with 60°C tap water (heat treatment alone, batch 1), 25°C tap water [neither heat nor high alkaline treatment, batch 2 (control)], 25°C wood ash extract (pH ≥ 11.0; high alkaline treatment alone, batch 3), and 60°C wood ash extract (both heat and high alkaline treatments, batch 4) at half volume (500 ml) for preparation. The next day, another half volume of 25°C wood ash extract (500 ml) was added to each batch (day 1). The wood ash extract was prepared by immersing 380 g wood ash (*Quercus phillyraeoides* A. Gray, Nagomi Co., Gobo, Wakayama, Japan) into 5 l tap water. The solution was boiled for 10 min and then the supernatant was extracted and used for the aiming temperature after the wood ash settled in the water. Triplicate batches were prepared to confirm the difference in the initiation of indigo reduction and changes in the dyeing intensity based on the initial preparation.

### Maintenance procedure of indigo fermentation and sampling

The fermentation batches were kept at 26°C in a temperature-regulated room and gently stirred once a day using a laboratory spoon with a top width of 30 mm, to prevent the localization of acid produced by the bacteria in the fermentation fluid. Wheat bran (1 g) was added on days 5, 19, 51, 85, and 194. The pH and redox potential (ORP) of the fermentation fluids were measured with a D-71 pH meter (Horiba, Kyoto, Japan) and a D-75 pH/ORP/CO meter (Horiba), respectively. The pH of the fermentation fluid was maintained between 9.67 and 11.2 using Ca(OH)_2_ to increase pH. The reducing state was tested by the dying ability of the fermentation fluid after dipping a small piece of cotton cloth into the fluid for 1 min and exposing it to air. After 2–5 min, the cloth was rinsed and dried. Dyeing intensity was measured by scanning the dyed cloth and analyzing the image using Mathematica (version 12.2). The intensity was expressed as L*a*b* value, which is the square root of L*^2^ + a*^2^ + b*^2^ in International Commission on Illumination and its System Colorimetry (CIE) L*a*b* color space where L*a*b* color space, L* represents lightness, and a* and b* represent chromaticity, which indicates hue and saturation, respectively. The extension of a* and b* indicates color direction as follows: a*, red; −a*, green; b*, yellow; and −b*, the blue. Before sampling, the fluid was stirred using a laboratory spoon to homogenize the fluid, and 700 μL aliquots were collected and stored in 35% (w/v) glycerol at −80°C until use.

### Illumina MiSeq sequencing

For DNA extraction, the FastDNA™ Spin kit for soil (MP Biomedicals, Santa Ana, CA, USA) was used according to the manufacturer’s instructions. The bacterial 16S rRNA gene sequence of the V3–V4 region (341F–805R) was amplified using the primer pair (5′-3′): V3V4f_MIX (ACACTCTTTCCCTACACGACGACGCTCTTCC-GATCT-NNNNN-CCTACGGG-NGGCWGCAG) and V3V4r_MIX (GTGACTGGAGTTCAGACGTG-TGCTCTTCCGATCT-NNNNN-GACTACHVGGGTATCTAATCC) purchased from Bioengineering Lab. Co. Ltd. (Sagamihara, Kanagawa, Japan). The primers consisted of an overhang necessary for the 2nd PCR, 0–5 bases of random sequences (described as N; adaptor), and a gene-specific sequence necessary for the amplification of 16S rRNA. The random sequences were used for quality control. The PCR solution (40 μL) consisted of 4 μL of 10× Ex buffer (TaKaRa Bio, Otsu, Shiga, Japan), 3.2 μL dNTPs (TaKaRa Bio), 2 μL each of the forward and reverse primers, 2 ng extracted DNA sample, and 0.4 μL of 5 U/ml Ex Taq polymerase (TaKaRa Bio). The cycling conditions were as follows: 94°C for 2 min; 25–35 cycles of 94°C for 30 s, 55°C for 30 s, and 72°C for 30 s; extension of 72°C for 5 min. The amplification products were purified using the QIAquick PCR Purification Kit (Qiagen, Mississauga, ON, Canada) following the manufacturer’s instructions. Product identity and quality were confirmed by agarose gel electrophoresis.

For next generation sequencing (NGS) analysis, the first PCR products were submitted to Bioengineering Lab. Co. Ltd. (Sagamihara, Kanagawa, Japan). The second PCR was performed with index-adapted primers to generate paired-end libraries (2 × 300 bp) for NGS using the MiSeq platform (Illumina, San Diego, CA, United States) and the MiSeq reagent kit v3 (Illumina).

### Sequencing data analysis

The 1st sequence primers and N-sequences were removed from the resulting gene sequences using Cutadapt version 1.18, the fastq files obtained from NGS were processed using QIIME2 ver. 2020.2 (Bolyen et al., 2019). Paired end read merge, quality control, and creation amplicon sequence variants (ASVs) were performed using DADA2 (Callahan et al., 2016). Taxonomic classification was conducted using the feature-classifier for the 16S rRNA gene sequence based on the primer pair of 341F–805R created based on the Silva database (Quast et al., 2013; Yilmaz et al., 2014). For updated taxonomic identification, representative sequences were used for a BLAST search in National Center for Biotechnology Information (NCBI^1^) (Miura et al., 2019). Rarefaction curves of observed Operational Taxonomic Units (OTUs) and Shannon index of diversity (Shannon, 1948) were plotted according to the QIIME2 alpha diversity script. Linear discriminant analysis (LDA) effect size (LEfSe) was conducted to identify the biomarkers for each batch by the Galaxy of Huttenhower Lab.^2^ Redundancy analysis (RDA) between the microbial community and environmental parameters was performed with R package (vegan ver. 2.6–4, ggrepel ver. 0.9.2, ggplot2 ver. 3.4.0, ggpubr ver. 0.5.0). Networks of the bacterial community based on the relative content change trend was analyzed as previously described (Tu et al., 2021). Predictive functions of the metagenomes were estimated using PICRUSt2 (Douglas et al., 2020). Based on the PICRUSt2 and QIIME2 results, taxa-functional relationships were analyzed using BURRITO (McNally et al., 2018). Correlation coefficient between changes in dyeing intensity of samples and corresponding changes in the intensity of the KEGG ortholog in “pred metagenome unstrat” file produced by the PICRUSt2 results was estimated.

## Results

### Changes in the microbial community during the early fermentation phase

From the 28 samples, 1,442,013 raw sequences were obtained, with an average of 51,500 sequences per sample (days 1–7) following fermentation preparation. After adapter trimming, and filtering for quality checking and chimeric exclusion, 687,015 sequences (average: 24,536 per sample) were obtained.

**Figure 1:**
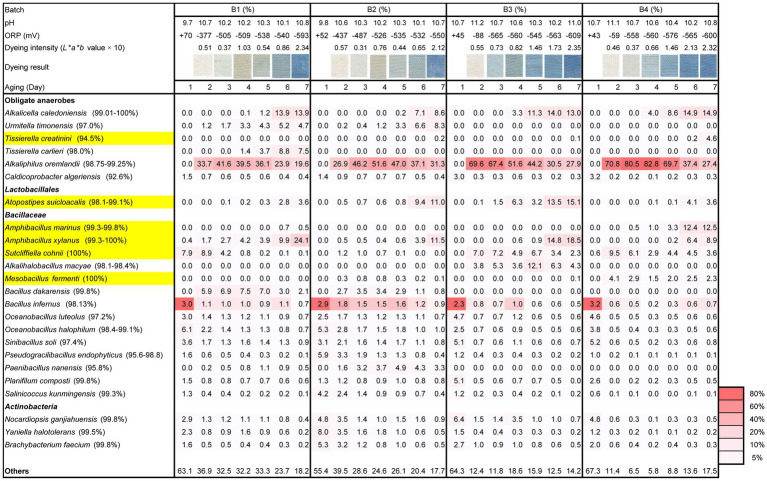
Changes in the relative abundance (%) of bacterial communities (≥ 4.1% in any sample) based on 16S rRNA analysis, dyeing intensity (dyeing result and L*a*b* value), ORP, and pH during days 1–7 following different initial treatments of *sukumo*. B1: batch 1 (*sukumo* treated with 60°C tap water); B2: batch 2 (*sukumo* treated with 25°C tap water [control]); B3: batch 3 (*sukumo* treated with 25°C wood ash extract); B4: batch 4 (control; *sukumo* treated with 60°C wood ash extract). Yellow marked taxa indicate the confirmed indigo-reducing taxa (including unpublished results). The percentages in the brackets indicate the similarities with the top hit taxa as revealed *via* a BLAST (blastn) search in NCBI.

Triplicate experiments confirmed the reproducibility of initiation of indigo reduction based on the treatment of *sukumo*. Microbiota analysis was performed with one sample set. Although wood ash treatment produced only approximately one unit difference in pH (batches 3 and 4; pH 10.7) compared with tap water treatment (batches 1 and 2; pH 9.7–9.8), contents of “others” were much higher in batches 1 and 2 than those in batches 3 and 4 after day 2. “Others” represents the sum of taxa present in less than 4.1% of all the samples (miscellaneous taxa), indicating that initial exposure of the microbial community in *sukumo* at approximately one unit higher than pH 9.7–9.8 strongly converged the microflora. However, the differences in the content of “others” were not observed between batches 1 and 2 on day 2, suggesting that heat treatment of 60°C of tap water was not very effective in converging the microflora. The lowest percentage of “others” (5.8%) and the highest percentage of the relative abundance of obligate anaerobic *Alkaliphilus oremalandii* (similarity: 98.8%–99.3%; abundance: 82.8%) in batch 4 at day 4 were observed (Figure 1). Moreover, the highest percentage of “others” (41.4%) and the lowest percentage of the relative abundance of *A*. *oremalandii* in batch 2 on day 2 were observed. The higher abundance (>50%) of *A*. *oremalandii* in batch 4 continued longer than in the other batches. These results suggest a strong relationship between the selection intensity of existing microorganisms in *sukumo* and relative abundance of obligate anaerobes in the initial treatment and the successive environmental changes.

The effect of the initial high pH treatment that was maintained was more pronounced in batches 2 and 4 than 1 and 2. However, the abundance of the alkaliphilic obligate anaerobic bacterium *A*. *oremalandii* was increased until days 3 and 4 in batches 3 and 4, respectively. In addition, the ratio of “others” decreased until day 4 in batch 4. Considering the ratio of “others,” which constitute miscellaneous low ratio constituent microorganisms, the converging effect for the microbial community was further enhanced in batches 1 and 2 by the addition of wheat bran on day 5.

Considering the rapid decrease in ORP, the remaining microorganisms in batches 1 and 2 after the preparation consumed oxygen more rapidly than those in batches 3 and 4 (Figure 1, Supplementary Figure S1A). Meanwhile, a pH of 11.2 in batch 3 and 11.1 in batch 4 deterred the metabolism of oxygen metabolizable microorganisms (Supplementary Figure S1B). However, the selected microorganisms of the initial treatments in batches 3 and 4 produced acid, which reduced the pH and allowed them to consume oxygen.

The earliest initiation of indigo reduction was observed in batch 3. The appearance of *Alkalihalobacillus macyae* (similarities: 98.1%–98.4%; content ratio: 5.3%) on day 3 in batch 3 was considered a contributing factor to the earlier indigo reduction. Initiation of indigo reduction was observed following the appearance of *Alkalicella caleodiniensis* (formerly *Proteinivoraceae*; 99.0%–100%; Tu et al., 2019b) in batches 1, 2, and 4. Indigo reduction was initiated on day 6 in batches 1 and 2. The appearance of *Tissierella* spp., *Amphibacillus* spp., and *Atopostipes* spp. may also contribute to the initiation of the indigo reduction. Although characterizations of these taxa were our unpublished results, these bacteria are indigo-reducing bacteria. This conversion effect by continuous high pH and low ORP was reflected in the ratio of “others” on day 7.

### Comparison of microbiota based on the heat or/and high pH treatment during days 2–5

**Figure 2:**
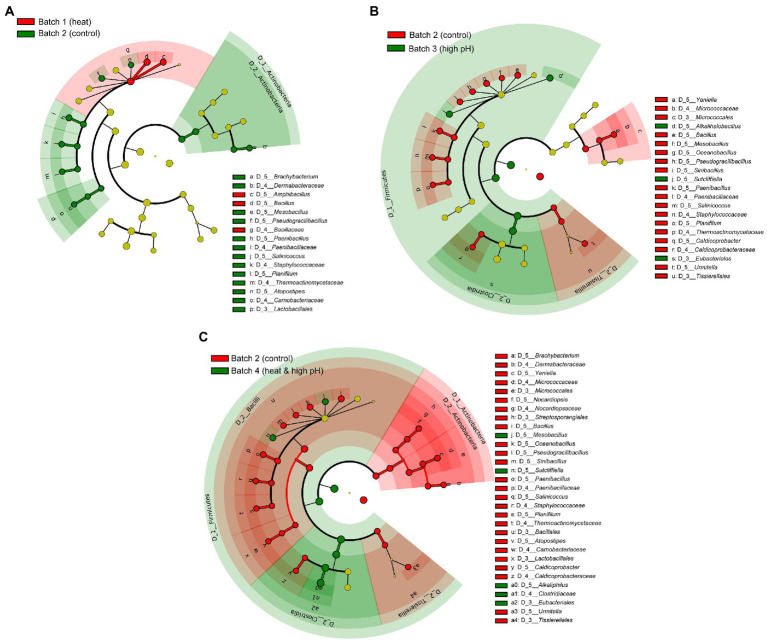
Bacterial markers of differently treated *sukumo* during days 2–5 following different initial treatments. **(A)** comparison between B1: batch 1 (*sukumo* treated with 60°C tap water) and B2: batch 2 (*sukumo* treated with 25°C tap water [control]). **(B)** comparison between B2 and B3: batch 3 (*sukumo* treated with 25°C wood ash extract) **(C)** comparison between B2 and B4: batch 4 (sukumo treated with 60°C wood ash extract). The LDA effect size (LEfSe) analysis was performed to identify the markers for each group (significant when LAD score > 3.6).

Differences in the microbiota due to treatment method of *sukumo* was most noticeable from day 2, when the effect of treatment began, prior to the effect of adding wheat bran, which appeared after day 6. Biomarker detection in each treatment batch was performed during days 2–5 (Figure 2). Heat treated (60°C) batch 1 compared with non-treated batch 2 showed that *Actinobacteria* (*Brachybacterium*), *Bacillaceae* (*Mesobacillus*, *Pseudogracillibacillus*, *Paenibacillus*, *Salinicoccus*, and *Planifilum*), and *Lactobacillales* (*Atopostipes*) were inhibited by treatment, while *Amphibacillus* (*Bacillaceae*) was enhanced (Figure 2A). High pH (pH ≥ 11.0) treated batch 3 compared with batch 2 showed that *Actinobacteria* (*Yaniella*), *Bacillaceae* (*Bacillus*, *Mesobacillus*, *Oceanobacillus*, *Pseudogracillibacillus*, *Sinibacillus*, *Paenibacillus*, *Salinicoccus*, *Planifilum* and *Thermoactinomyce*), and *Caldicoprobacteraceae* (*Caldicoprobacter*) were inhibited by the alkaline treatment, while *Bacillaceae* (*Alkalihalobacillus* and *Sutcliffiella*) and *Eubacteriales* were enhanced (Figure 2B). Heat and high pH treated batch 4 compared with batch 2 showed wider range of inhibited taxa compared with alkaline treatment (Figure 2C). Indeed, *Actinobacteria* (*Brachybacterium* and *Nocardiopsis*), *Bacillaceae* (*Paenibacillus*) and *Lactobacillales* (*Atopostipes*) were further inhibited compared with alkaline treatment alone; meanwhile, *Mesobacillus*, S*utcliffiella*, and *Eubacteriales* (*Alkaliphilus*) were enhanced by the treatment. Therefore, high pH treatment had larger impact than heat treatment on microbiota, while heat combined with high pH treatment showed the biggest impact on microbiota among the three treatments.

### Changes in the microbial community in the stable and aged states of fermentation

Since batches 1 and 2 showed similar changes in their microbiota on 7, we focused on batches 2, 3 and 4 from days 10–209 for subsequent microbiota analysis. A total of 873,986 raw sequences were obtained from a total of 18 samples (average: 48,555/sample), one from each day (days 10–209) after fermentation preparation. After adapter trimming, filtering for quality checking, and chimeric exclusion, 353,688 sequences (average: 19,649/sample) were obtained.

The batches exhibited clear dyeing on day 7 and further strong dyeing on day 10. This may be attributed to the effect of the maintained high pH from day 2, low ORP, and the introduction of wheat bran on day 5 across all batches. Consequently, all three batches exhibited relatively strong dyeing intensity and the constituents in the microbiota were relatively similar after day 10, indicating that all the batches reached a stable state for indigo reduction in fermentation, regardless of the initial treatment.

**Figure 3:**
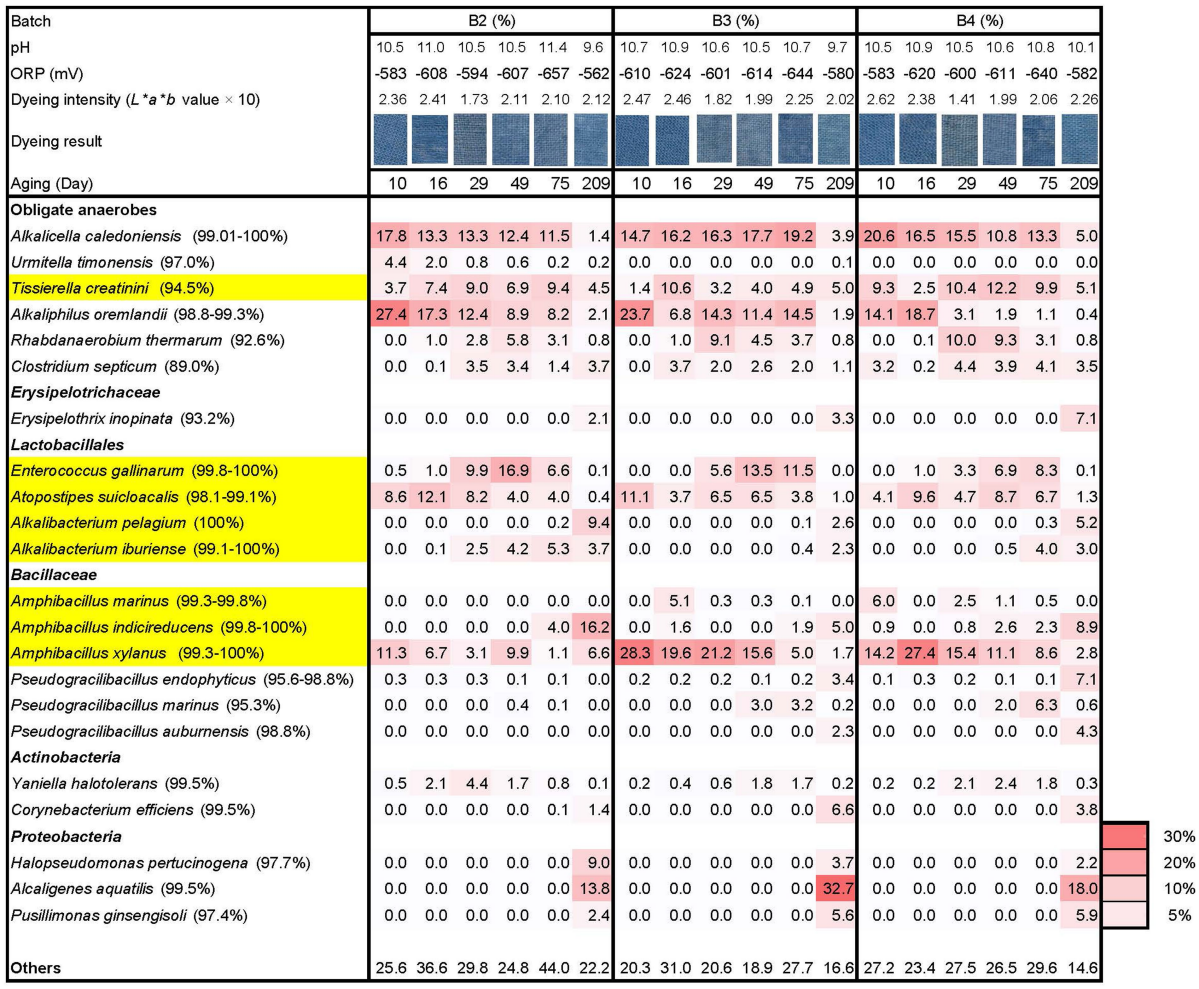
Changes in the relative abundance (%) of bacterial communities (≥ 4.1% in any sample) based on 16S rRNA analysis, dyeing intensity (dyeing result and L*a*b* value), ORP, and pH during days 10–209 following different initial treatments of *sukumo*. B2: batch 2 (*sukumo* treated with 25°C tap water [control]); B3: batch 3 (*sukumo* treated with 25°C wood ash extract); B4: batch 4 (*sukumo* treated with 60°C wood ash extract). Yellow marked taxa indicate the confirmed indigo-reducing taxa (including unpublished results). The percentages in the brackets indicate the similarities with the top hit taxa as revealed *via* a BLAST (blastn) search in NCBI.

Although no strong selection was observed in batch 2 on day 0 compared with the other batches, the microbiota converged appropriately on day 7 due to the continuously high pH starting on day 2 with the addition of Ca(OH)_2_, low ORP and wheat bran. Furthermore, the establishment of desirable microbiota was observed on day 10, and relatively similar microbiota among the batches was observed on day 75 in this batch (Figure 3). The microbiota in batch 2 were mainly comprised of *A*. *caledoniensis* (similarity: 99.0%–100%; abundance ratio: 11.5%–17.8% on days 10–75), *A*. *oremlandii* (similarity: 98.8%–99.3%; abundance ratio: 8.2%–27.4% on days 10–75), *Enterococcus gallinarum* (similarity: 99.8%–100%; abundance ratio: 6.6%–16.9% on days 29–75), *Atopostipes suicloacalis* (similarity: 98.1%–99.1%; abundance ratio: 4.0%–12.1% on days 10–49) and *Amphibacillus xylanus* (similarity: 99.3%–100%; abundance ratio: 3.1–11.3 on days 10–49). The microbiota of batch 2 was different with regards to the lower abundance ratio of *A*. *xylanus*, the earlier appearance of *Alkalibacterium iburiense* and the higher ratio of “others.”

The microbiota of batch 3 was relatively similar to that of batch 4. However, the present ratio in *Tissierella creatini* (similarity: 94.5%) to *A*. *oremalandii* (similarity: 98.8%–99.3%) differed in the taxa comprising obligate anaerobes. Moreover, the ratio in the fluctuations of *A*. *xylanus* (similarity: 99.3%–100%) was also different.

On day 209, all three batches contained *Proteobacteria* in their microbiota, which comprised more than 20% in the microbiota ratio suggesting that although *sukumo* introduced Gram-positive bacteria into the fermentation system. However, Gram-negative bacteria exhibited superior adaptation ability compared with Gram-positive bacteria in the liquid environments. In addition, the ratio in *Alkalibacterium* spp. was increased, while other taxa existed categorized in *Lactobacillales* [e.g., *Atopostipes suicloacalis* and *Enterococcus gallinarum* (99.8%–100%)] were decreased. In the obligate anaerobes category, the ratio of preferred taxa on day 75 (e.g., *Alkakucella caledoniensis* and *Alkaliphilus oremlandii*) was decreased and facultative anaerobic *Erysipelotrichaceae* (93.2% similarity with *Erysipelothrix inopinate*) and *Corynebacterium efficiens* (similarity: 99.5%) appeared.

### Changes in alpha diversities

Changes in alpha diversity at a sampling depth of 8,825 reads from the rarefaction curves based on the observed OTUs and Shannon index of the four batches are shown in Supplementary Figure S2. High alkaline treated batches 3 and 4 showed significant decreases in both the observed OTUs and Shannon index from days 1 to 2. Moreover, the decrease in these values by heat (60°C) treated batch 1 was not as intense as a high alkaline treatment (batches 3 and 4). Although the high pH treatment (batch 3) and both high pH and heat treatment (batch 4) were performed at the preparation of fermentation (day 0), the decrease in the diversity of microbial constituents continued until days 2 and 4, respectively. After the decrease in diversity, recovery was observed on days 3 and 5 in batches 3 and 4, respectively.

Although the Shannon index of batches 1 and 2 did not changed the diversity from days 3–7, the observed OTUs gradually decreased. Furthermore, the introduction of wheat brane caused a further decrease in the observed OTUs. Surprisingly, all the alpha-diversity of the four batches, except for observed OTUs in batch 1, converged to almost the same values. These results are comparable with the similar dyeing intensity among the four batches and the ratio changes of the “others.”

### Identification of environmental factors influencing the convergence of microbiota to indigo reducing state

**Figure 4:**
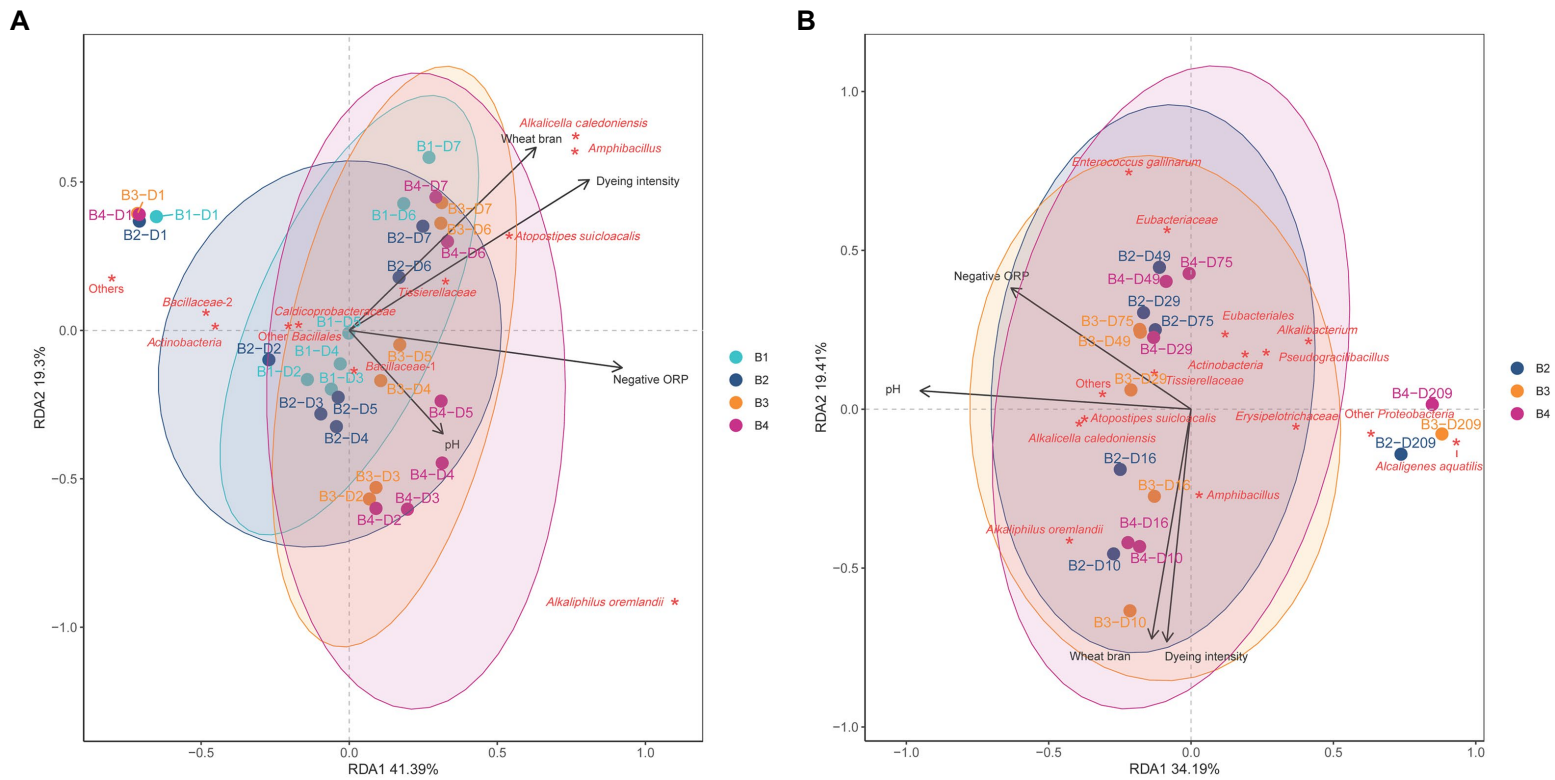
Redundancy analysis (RDA) of indigo fermentations during days 1–7 **(A)** and days 10–209 **(B)** following different initial treatments of *sukumo*. B1: batch 1 (*sukumo* treated with 60°C tap water); B2: batch 2 (*sukumo* treated with 25°C tap water [control]); B3: batch 3 (*sukumo* treated with 25°C wood ash extract); B4: batch 4 (*sukumo* treated with 60°C wood ash extract). The labels for samples indicate the batch number and the number of days of fermentation (B, batch; D, day). Red asterisks represent the core microbiota. Black arrows indicate different environmental factor. Wheat bran was considered an environmental factor given that it would be consumed within 1 month. Percentages on the axes represent the eigenvalues of principal components. **(A)** *Bacillaceae*-1 contains *Sutcliffiella cohnii*, *Alkalihalobacillus macyae*, *Mesobacillus fermenti*, and *Bacillus dakarensis*; *Bacillaceae*-2 contain *Oceanobacillus*, *Sinibacillus*, and *Pseudogracilibacillus*; Other *Bacillales* contains *Paenibacillus* and *Salinicoccus kunmingensis*. **(B)** *Eubacteriaceae*: *Rhabdanaerobium* *thermarum* (92.6%); *Eubacteriales*: *Clostridium septicum* (89.0%).

The relationship between the changes in microbial communities among the three batches (batches 2–4) and the influence of environmental parameters were estimated using RDA (Figures 4A,B). The high impact of hot wood ash extract in batch 4 on microbiota was reflected in the distance between day 1 and after day 2 (Figure 4A). Meanwhile, the higher impact of the high pH in batch 3 compared with the heat treatment in batch 1 was reflected by the distances between days 1 and 2. Changes in microbiota of batch 1 was very similar that of the non-heat-treated batch 2. High pH treatment (batches 3 and 4) produced more rapid transitional changes from days 1 to 2. The microbiota treated with high pH was changed and influenced by low ORP from day 2. However, susceptibility to low ORP decreased in batches 1 and 2 compared with 3 and 4. The changing velocity of each microbiota was lower during days 2–5 compared with until day 2. However, the velocity became increased by the wheat bran introduced at day 5. Although the microbiota of each batch was differed, the wheat bran strongly shifted the microbiota towered indigo reducing state from day 5. The contribution to indigo reducing state remains unknown, however the appearance of *Alkaliphilus oremlandii* is strongly linked to the high pH and low ORP. Furthermore, *Tissierellaceae*, *Atopostipes suicloacalis*, *Amphibacillus* and *Alkalicella caledoniensis* are core members at days 6–7 (Figure 4A). Batch 3 started indigo reduction on day 3, which was attributed to the contribution of *Bacillaceae*-1. Although the transitional change from days 1 to 5 of each batch differed, all day 7 positions were similar. This is comparable with the changes in the Shannon index of alpha diversity in the 4 batches converged to nearly the same values on day 7.

### Relationship between changes in microbiota after day 10, environmental parameters, and related microorganisms

All batches exhibited similar changing tendencies from day 10 compared with the changes in days 1–7 (Figure 4B). The changing direction was the same with similar positioning of microbiota. Figure 4B shows that the change rate of microbiota was low based on the slow decomposing substrate, wheat bran, while the continuous introduction of the substrate is strongly associated with the maintenance of the dyeing intensity during the aging of each microbiota. However, the dyeing intensity gradually decreased with aging during fermentation. During the convergence of each microbiota toward an aging state, successive changes in several functional-redundant-indigo-reducing different taxa appeared and sustained indigo reduction for a long duration. Around day 29, *Alkaliphilus oremlandii*, *Amphbacillus Atopstipes suicloacalis*, and *Alcalicela caledoniensi*s were the core bacteria that sustained this ecosystem. However, after day 29, in addition to *Atopstipes suicloacalis*, and *Alcalicela caledoniensi*s, *Tissierellaceae* and *Alkalibacterium* were the core members in the microbiota. Meanwhile, after day 75, *Eubacteriaceae*, *Eubacteriales*, and *Enterococcus gallinarum* were added as the core members in the ecosystem, and at day 209, Gram-negative bacteria became the core members of the microbiota. The isolated positioning of the microbiota in this series of change to day 209 was in the opposite to the high pH, possibly due to local changes in pH of the fermentation fluid.

### Microbial interaction network

**Figure 5:**
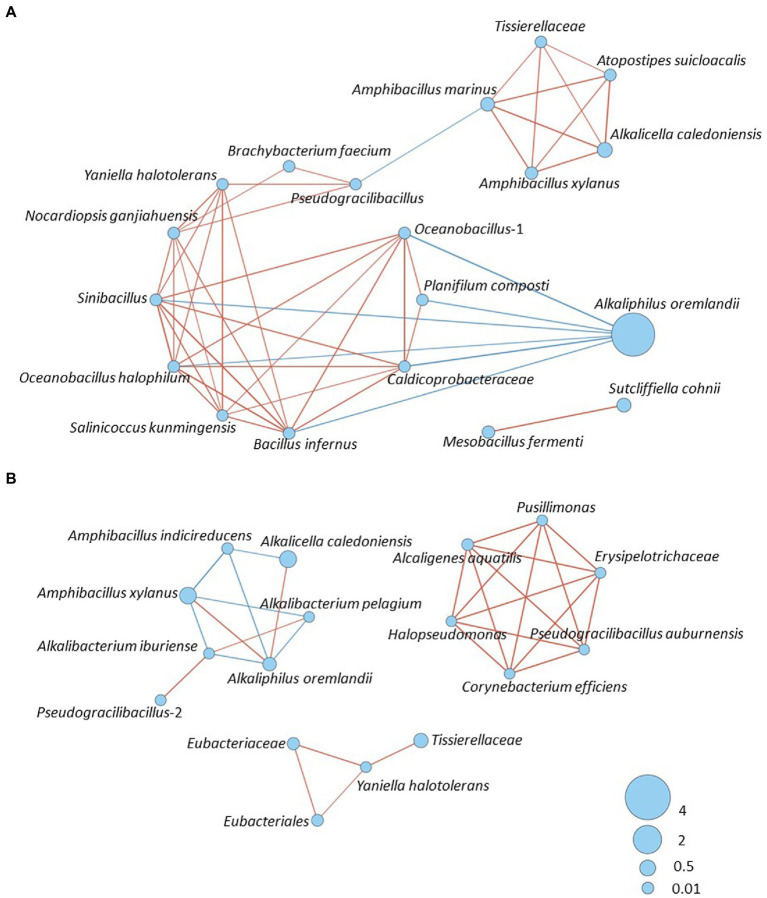
Bacterial community networks of the bacterial community based on the relative content change trend analyzed with Speaman’s rank correlation coefficient [*rs* > 0.6, *p* < 0.05]; **(A)** Batch 4, days 1–7. **(B)** Batch 4, days 10–209. The red and blue lines represent positive and negative correlation, respectively. The thickness of the lines corresponds to the strength of the relationship (0.6 < *rs* ≤ 1), while circle size shows accumulated taxon abundances during each fermentation period.

We analyzed the interaction of existing bacteria on days 1–7 (Figure 5A) and 10–209 (Figure 5B) and found insignificance differences in the detail between the batches, with similar tendencies. Therefore, the results from batch 4 are presented as the representative. Firstly, a relatively strong network was constructed based on the survived oxygen-metabolizing taxa such the 3 taxa of *Actinobacteria* and *Sinibacillus*, and 2 taxa of *Oceanobacillus*, *Salinicoccus kunmingensis*, *Bacillus infernus*, *Planifilum composti* and *Caldicoprobacteraceae*. This initial network was destroyed by the high pH and the low ORP. Subsequently, *Msobacillus fermenti*, *Sutcliffiella cohnii* and *Alkaliphilus oremlandii* appeared. Toward an enhanced indigo reducing state at day 7, network consisted of 2 taxa of *Amphibacillus*, *Tissierellaceae*, *Atopstipes suicloacalis*, and *Alcalicela caledoniensi*s was constructed. After day 10, the similar network, constructed until day 7, was succeeded; however, the inter positive relationship had weakened. The gravity of the network gradually moved to the latter stage as shown in Figure 5B. Networks containing Gram-negative bacteria established after day 75 were relatively strong.

### Functional abundance for the initiation of indigo reduction

The predictive function of the metagenomes was estimated to clarify the prerequisite functional abundances for the initiation of indigo reduction. Since the initiation of indigo reduction was observed only in batch 3 on day 3, the functional abundance ratio between batch 3 and the other batches was estimated. The ratio in the functional subpathways containing a ratio > 1.05 in batch 3(B3)/batches 1(B1), B3/batch 2 (B2), or B3/batch 4 (B4) on day 3 are listed in Supplementary Figure S3. Among the presented subpathways, “phosphotransferase system (PTS)” and “starch and sucrose metabolisms” followed by “Prokaryotic defense system” and “nicotinate and nicotinamide metabolism” were identified. In addition, although their ratios are not very high, the subpathways related to the reconstitution of cellular functions and metabolism related to electron carriers and motility were also found.

Although the dyeing intensities during the initiation days were not always strong, batches 2–4 exhibited strong dyeing intensities compared with day 10. To understand the prerequisite functional subpathways that initiate dyeing, the functional abundance ratios of day 10 (beginning of intense dyeing)/day 2 (before initiation of dyeing) was estimated. In addition, ratio of day 16 (stable phase in intense dyeing)/day 2 in batches 2–4 were estimated (Supplementary Figure S4). To understand the important functions for enhancing dyeing intensity, the functional abundance ratios of day 10/day at the initiation of dyeing and day 16/ day at the initiation of dyeing in batches 2–4 were estimated (Supplementary Figure S4). The listed categories and super pathways were similar to the list in Supplementary Figure S3, except for the subpathways in super pathways of cell motility and metabolism (category of unclassified). Subpathways in the category of “unclassified” are important based on the subpathway in either batch 2 or 4. Increased items from Supplementary Figure S3 were reflected in the diversity change in the microorganisms related to indigo reduction. Although the listed subpathways were increased, it was considered that the “phosphotransferase system (PTS)” and “starch and sucrose metabolisms” remain important for the initiation of dyeing and enhancement of the dyeing intensity. In addition, although their ratios were lower in the two subpathways, “Prokaryotic defense system” and “nicotinate and nicotinamide metabolism” were relatively high.

The functional abundance of the subpathways, which were picked up in Supplementary Figure S4 and the contribution of the subpathways by major constituted taxa were estimated (Supplementary Figure S5) on day 3. The initial fermentation stage in batch 3 could be explained by the functional abundances of the subpathways of “phosphotransferase system (PTS)” and “starch and sucrose metabolisms.” The specific abundancies in batch 3 were attributed to the genus *Alkalihalobacterium*, which specifically existed in this batch. This genus exhibited a higher contribution than other taxa considering the existing abundance ratio. The contribution for the listed subpathways by the genus *Alkaliphilus* was relatively low considering the existing ratio. However, this genus contributed to “amino acid metabolisms” rather than that of the carbohydrates.

**Figure 6:**
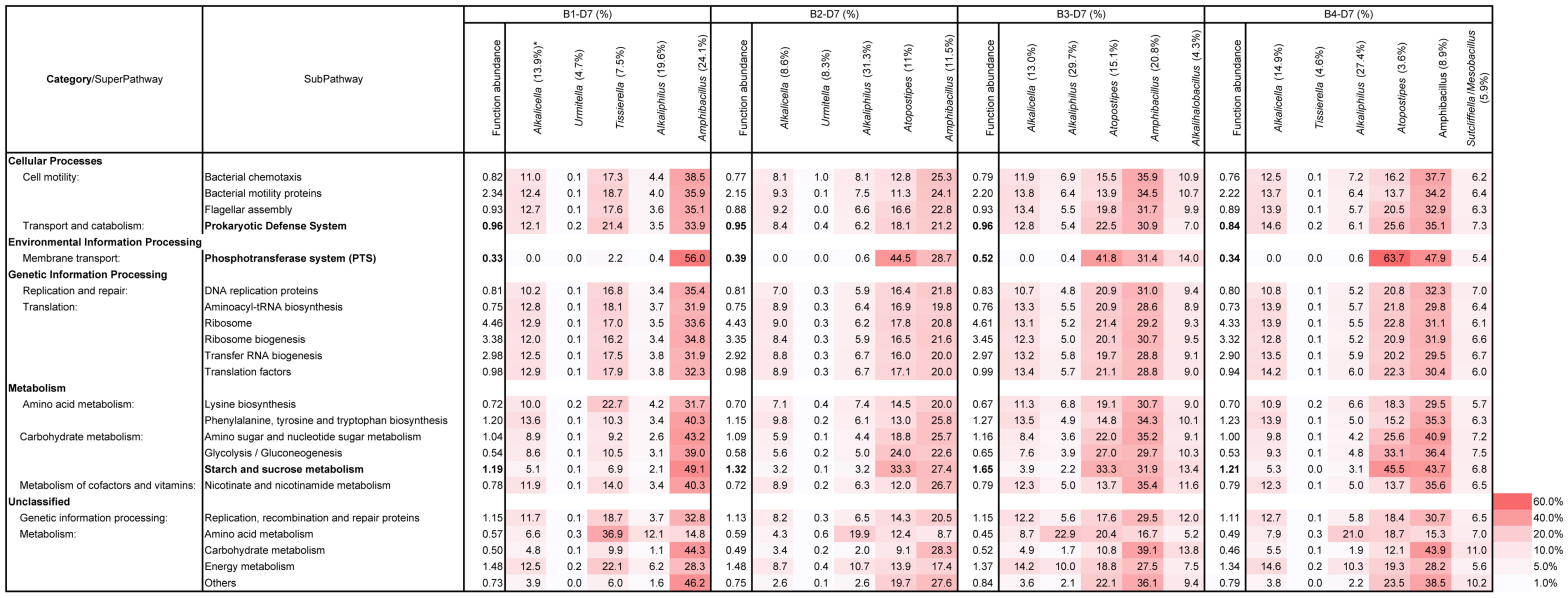
Functional abundance ratio (%) of the total subpathways and the contributing ratio (%) of the major constituent taxa in the subpathways related to the initiation and enhancement of indigo reduction at D7 samples in B1–4. *: The number in the parentheses is the existence ratio (%) in the microbiota. The metagenomic prediction produced using PICRUSt2 and BURRITO is shown. The important subpathways and functional abundance ratios are indicated in bold letters. B, batch; D, day.

The time from the preparation (day 0) to the initiation of indigo dyeing was different depending on the initial preparation. However, a moderate and similar intensity of dyeing was observed on day 7 in all batches (Figure 1). All four batches exhibited high functional abundance ratios in the three subpathways of “phosphotransferase system (PTS)” and “starch and sucrose metabolisms” (Figure 6). These findings consistent with the dyeing results. Taxa that occupied major constituents were increased as the abundance ratio of *Alkaliphilus* was decreased in all the batches. The functional abundance of subpathways by genera *Amphibacullus* and/or *Atopstipes* contributed to fulfilling the abundance of the important subpathways involved in indigo dyeing. The contributions of subpathways important for dyeing in obligate anaerobes (e.g., *Alkaliphilus* and *Umitella*) were relatively low considering the present ratio. However, the contribution by genus *Tissierella* in batch was high considering the existing ratio. These obligate anaerobes most likely did not significantly contribute to the metabolisms of carbohydrates in this ecosystem.

### Functional abundance in the stable and aged states of the fermentation

From days 7 to 10, the dyeing intensity increased; however, no significant change was found in the ratio of the functional abundance ratio, which was important for indigo reduction (Figure 6 and Supplementary Figure S6). The increase in staining intensity was likely due to the qualitatively improved metabolic function of the microbiota rather than a quantitative increase in abundance. The decrease in the ratio of *Alkaliphilus* and increase in the ratio of *Amphibacillus* and/or *Alcalicella* and *Tissierella* were improved during days 7–10. The former exhibited a relatively low contribution for the important subpathways for indigo reduction, while the latter exhibited relatively high contribution for the important subpathways for indigo reduction (Supplementary Figure S6). In this way, important functions for the reduction of indigo and material circulation in the system shifted to the main constituent taxa on day 10.

**Figure 7:**
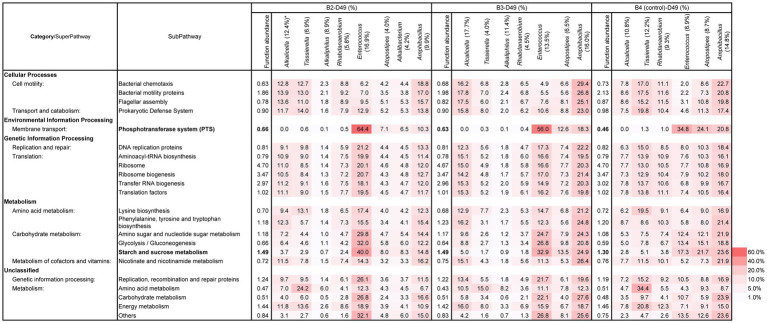
Functional abundance ratio (%) of the total subpathways and the contributing ratio (%) of the major constituent taxa in the subpathways related to the initiation and enhancement of indigo reduction at D49 samples in B2–4B. *: The number in the parentheses is the existence ratio (%) in the microbiota. The metagenomic prediction produced using PICRUSt2 and BURRITO is shown. There important subpathways and the functional abundance ratios are indicated in bold letters. B, batch; D, day.

After day 10, the functional abundance ratio of the subpathways of “phosphotransferase system (PTS)” and “starch and sucrose metabolisms” remained relatively high. The established effective metabolic pathway was maintained after day 10. The contribution of the subpathways by major taxa was also estimated in the microbiota at day 49 (Figure 7). The highly contributed taxa for the important subpathways were different based on the batch. *Alcalicella*, *Tissierella*, *Enterococcus*, and *Ampibacillus* were the major contributors to the important subpathways in batch 2. Meanwhile, *Alcalicella*, *Enterococcus*, and *Amphibacillus* were the major contributors to the indigo reduction in batch 3.

### KEGG ortholog related to the dyeing intensity and initiation of indigo reduction

Table 1 lists the KEGG orthology correlated with staining intensity, which shows the correlation coefficients of ≥ 0.69 selected up from KEGG orthology items of 5,458 described in “pred metagenome unstrat” file created with PICRUSt2. Although the functional category differed from the results of the BURRITO analysis, the functions described the “phosphotransferase system (PTS),” “starch and sucrose metabolisms,” “Prokaryotic defense system,” and “nicotinate and nicotinamide metabolism” were found in the list. Among the KEGG orthology items, seven NAD(P)-dependent oxidoreductases were selected and the related taxa in each orthology and ratio “CountContributedByOTU” at the initiation of indigo reduction in batches 3 [from days 2 to 3 (D3/D2)] and 4 was [from days 3 to 4 (D4/D3)] were estimated using the “pred metagenome contrib.legacy” produced by PICRUSt2 analysis (Table 2). Enhancement of H^+^-translocating NAD(P) transhydrogenase subunit beta [EC:1.6.1.2 7.1.1.1] (K00324, K00325) of *Alkalihalobacillus macyae* and NADP-dependent alcohol dehydrogenase [EC:1.1.1.2], uronate dehydrogenase [EC:1.1.1.203], and D-lactate dehydrogenase [EC:1.1.1.28] of *Atopostipes suicloacalis* caused the initiation of indigo reduction in batch 3 (Table 2). Meanwhile, enhancement of formylmethanofuran dehydrogenase subunit E [EC:1.2.7.12] and anaerobic carbon-monoxide dehydrogenase catalytic subunit [EC:1.2.7.4] of *Alkalicella caledoniensis* might have contributed to the initiation of indigo reduction in batch 4 (Table 2). In batches 1 and 2, changes toward the indigo reduction are attributed to the increase in the ratio of *Atopostipes suicloacalis* and *Alkalicella caledoniensis* (Figure 1).

**Table 1:** KEGG orthologies predicted by PICRUSt2 correlated with changes in dyeing intensity (correlation coefficient ≥ 0.69).

KEGG orthology	Correlation coefficient	Symbol	Description	Function
K08977	0.79	cruF	bisanhydrobacterioruberin hydratase [EC:4.2.1.161]	Metabolism of terpenoids and polyketides; Carotenoid biosynthesis
K02538	0.79	manR	mannose operon transcriptional activator	Protein families: genetic information processing; Transcription factors
K16788	0.76	niaX	niacin transporter	Protein families: signaling and cellular processes; Transporters
K03973	0.76	pspC	phage shock protein C	Protein families: signaling and cellular processes;**Prokaryotic defense system**
K01224	0.76	E3.2.1.89	arabinogalactan endo-1,4-beta-galactosidase [EC:3.2.1.89]	Unclassified: metabolism; Enzymes with EC numbers
**K00325**	0.76	pntB	H^+^-translocating NAD(P) transhydrogenase subunit beta [EC:1.6.1.2 7.1.1.1]	Metabolism of cofactors and vitamins;**Nicotinate and nicotinamide metabolism**
K02791	0.75	malX	maltose/glucose **PTS system** EIICB component [EC:2.7.1.199 2.7.1.208]	Amino sugar and nucleotide sugar metabolism; Metabolic pathways
K11646	0.75	K11646	3-dehydroquinate synthase II [EC:1.4.1.24]	Amino acid metabolism; Phenylalanine, tyrosine and tryptophan biosynthesis
K14660	0.75	nodE	nodulation protein E [EC:2.3.1.-]	Unclassified: metabolism; Enzymes with EC numbers
**K00324**	0.73	pntA	H^+^-translocating NAD(P) transhydrogenase subunit alpha [EC:1.6.1.2 7.1.1.1]	Metabolism of cofactors and vitamins;**Nicotinate and nicotinamide metabolism**
K03339	0.73	iolJ	6-phospho-5-dehydro-2-deoxy-D-gluconate aldolase [EC:4.1.2.29]	**Carbohydrate metabolism**; Inositol phosphate metabolism
K09163	0.72	K09163	uncharacterized protein	Poorly characterized; Function unknown
K00197	0.72	cdhE, acsC	acetyl-CoA decarbonylase/synthase, CODH/ACS complex subunit gamma [EC:2.1.1.245]	Metabolic pathways; Microbial metabolism in diverse environments
K15023	0.72	acsE	5-methyltetrahydrofolate corrinoid/iron sulfur protein methyltransferase [EC:2.1.1.258]	Metabolic pathways; Microbial metabolism in diverse environments
**K11261**	0.72	fwdE, fmdE	formylmethanofuran dehydrogenase subunit E [EC:1.2.7.12]	Metabolic pathways; Microbial metabolism in diverse environments
K03389	0.72	hdrB2	heterodisulfide reductase subunit B2 [EC:1.8.7.3 1.8.98.4 1.8.98.5 1.8.98.6]	Metabolic pathways; Microbial metabolism in diverse environments
**K00198**	0.71	cooS, acsA	anaerobic carbon-monoxide dehydrogenase catalytic subunit [EC:1.2.7.4]	Metabolic pathways; Microbial metabolism in diverse environments
K15051	0.71	endA	DNA-entry nuclease	Unclassified: signaling and cellular processes; Unclassified viral proteins
K02173	0.71	yggC	(RefSeq) P-loop NTPase domain-containing protein YggC K02173 putative kinase	Unclassified: metabolism; Cofactor metabolism
K02750	0.71	glvC, malP, aglA	alpha-glucoside**PTS system** EIICB component [EC:2.7.1.208 2.7.1.-]	**Carbohydrate metabolism**;**Starch and sucrose metabolism**
K13677	0.71	dgs, bgsA	1,2-diacylglycerol-3-alpha-glucose alpha-1,2-glucosyltransferase [EC:2.4.1.208]	Lipid metabolism; Glycerolipid metabolism
K00105	0.71	E1.1.3.21	alpha-glycerophosphate oxidase [EC:1.1.3.21]	Lipid metabolism; Glycerolipid metabolism
K02530	0.71	lacR	DeoR family transcriptional regulator, lactose phosphotransferase system repressor	Protein families: genetic information processing; Transcription factors
**K08325**	0.71	yqhD	NADP-dependent alcohol dehydrogenase [EC:1.1.1.2]	**Carbohydrate metabolism**; Propanoate metabolism
K10530	0.71	lctO	L-lactate oxidase [EC:1.1.3.2]	Unclassified: metabolism; Enzymes with EC numbers
K18217	0.71	steB, tetB46	ATP-binding cassette, subfamily B, tetracycline resistant protein	Membrane transport; ABC transporters
K09952	0.71	csn1, cas9	CRISPR-associated endonuclease Csn1 [EC:3.1.-.-]	Protein families: signaling and cellular processes;**Prokaryotic defense system**
K03390	0.71	hdrC2	heterodisulfide reductase subunit C2 [EC:1.8.7.3 1.8.98.4 1.8.98.5 1.8.98.6]	Energy metabolism; Methane metabolism
K02291	0.71	crtB	15-cis-phytoene synthase [EC:2.5.1.32]	Metabolism of terpenoids and polyketides; Carotenoid biosynthesis
K01215	0.71	dexB	glucan 1,6-alpha-glucosidase [EC:3.2.1.70]	Unclassified: metabolism; Enzymes with EC numbers
K06896	0.71	mapP	maltose 6′-phosphate phosphatase [EC:3.1.3.90]	**Carbohydrate metabolism**;**Starch and sucrose metabolism**
K02779	0.71	ptsG	glucose**PTS system** EIICB or EIICBA component [EC:2.7.1.199]	**Carbohydrate metabolism**; Glycolysis / Gluconeogenesis
K01071	0.71	MCH	medium-chain acyl-[acyl-carrier-protein] hydrolase [EC:3.1.2.21]	Lipid metabolis; Fatty acid biosynthesis
K09758	0.71	asdA	aspartate 4-decarboxylase [EC:4.1.1.12]	Amino acid metabolism; Alanine, aspartate and glutamate metabolism
K02781	0.71	srlB	glucitol/sorbitol**PTS system** EIIA component [EC:2.7.1.198]	**Carbohydrate metabolism**; Fructose and mannose metabolism
K07078	0.71	FRM2, YCLX08C	(RefSeq) type II nitroreductase	Poorly characterized; Function unknown
K03388	0.71	hdrA2	heterodisulfide reductase subunit A2 [EC:1.8.7.3 1.8.98.4 1.8.98.5 1.8.98.6]	Energy metabolism; Methane metabolism
**K18981**	0.70	udh	uronate dehydrogenase [EC:1.1.1.203]	**Carbohydrate metabolism**; Ascorbate and aldarate metabolism
K15780	0.70	tilS-hprT	bifunctional protein TilS/HprT [EC:6.3.4.19 2.4.2.8]	Nucleotide metabolism; Purine metabolism
**K03778**	0.70	ldhA	D-lactate dehydrogenase [EC:1.1.1.28]	**Carbohydrate metabolism**; Pyruvate metabolism
K02769	0.69	fruAb	fructose**PTS system** EIIB component [EC:2.7.1.202]	**Carbohydrate metabolism**; Fructose and mannose metabolism
K01669	0.69	phr, PHR1	deoxyribodipyrimidine photo-lyase [EC:4.1.99.3]	Protein families: genetic information processing; DNA repair and recombination proteins
K02793	0.69	manXa	mannose**PTS system** EIIA component [EC:2.7.1.191]	**Carbohydrate metabolism**; Fructose and mannose metabolism

Bold letters in Description and Function exhibit referred with the results of BRRITO. Items exhibit bold letter in KEGG orthology are candidate enzymes directly related with extracellular electron transportation. K00198 and K11261: related to obligate anaerobes; K03778, K08325 and K18981: related to lactic acid bacteria; K00324 and K00325; related to facultative anaerobes.

**Table 2:** The KEGG orthologies belonging to the NAD(P)-dependent oxidoreductase that exhibited.

KEGG orthology	Taxon identified from DNA sequencing of OTU	Ratio in D3/D2 in CountContributedByOTU
K08325, K03778, K18981		
	*Atopostipes suicloacalis* (99.1%)	11.0
	*Atopostipes suicloacalis* (99.1%)	9.1
K00324, K00325		
	*Alkalihalobacillus macyae* (98.1%)	1.4
		Ratio in D4/D3 in CountContributedByOTU
K00198, K11261		
	*Alkalicella caledoniensis* (99.3%)	88.1

Highly correlated with the initiation of indigo reduction in batch 3 in day 3/day 2 (D3/D2) and batch 4 in day 4/day 3(D4/D3) in Table 1.

## Discussion

An initial treatment of *sukumo* with hot wood ash extract (60°C–80°C, pH > 10.5) has been conventionally used in indigo fermentation for reducing indigo. However, the environmental effects on the microbiota during this treatment, as well as the mechanisms underlying the microbial succession toward stable state remain unknown. Subsequently, wheat bran is added to the fermentation fluid as a substrate for the microorganisms, but the timing of this treatment varies depending on the craftsperson. We tested the effect of pH and heat treatment on *sukumo* and found that a high pH has larger impact on the microbiota in *sukumo* than a high temperature when selecting for microorganisms that contribute to indigo reduction. In addition, the microbiota in the *sukumo* treated with high pH exhibited more rapid change than those of non-alkaline treated batches. In the initial state of the fermentation (days 2–5), the different microbial communities with different dyeing intensities appropriately converged to facilitate indigo reduction on day 7 by the effect of wheat bran added at day 5. This convergence is occurred based on the maintenance of a high pH (day 1~) and low redox potential (day 2~) and the introduction of wheat bran at day 5. Indigo reducing functions of the microbiota were initiated or enhanced concomitant with major substrate changes from readily utilizable substrates derived from *sukumo* and dead microbial cells resulting from transitional changes of microbiota to hardly utilizable substrates (wheat bran or cellulose and xylan in *sukumo*) (Lopes et al., 2021). Wheat bran was not added in the early fermentation phase in most of our previous experimental batches, and the microbiota did not reach to the maturation phase within day 30 and a decrease in staining intensity was observed around day 56 (Tu et al., 2021). The present study showed that the addition of wheat bran in the early fermentation stage led to a stable state of indigo reduction (day 10) in all the fermentation batches tested. Introducing the carbohydrate as a source of energy for microbiota also increased dyeing intensity. Finally, the microbiota maintained their indigo reducing function in the closed ecosystem as the wheat bran was the sole external nutrient and changed the slowly circling substances involved in the high pH conditions. This stable stage is similar to the maturation in the general fermentation process. During the maturation period of indigo reduction fermentation, circulating substances in the ecosystem and indigo reduction function are synchronized.

Considering the relationship between the predicted functional abundance based on the microbial community and the differences in the dyeing results attributed to differences in the preparation procedures, fulfillment of the “phosphotransferease system (PTS)” and “starch and sucrose metabolism” subpathways helped to increase the dyeing intensity and the initiation of indigo reduction. In addition to the two main subpathways, other enhanced subpathways related to the indigo reduction included: carbohydrate metabolisms, accession for the substrates, reconstitution of cellular functions, defense for bacteriophage and antibiotics, and metabolism related to electron carriers. These functions indicate that indigo reduction occurs when the bacteria with indigo-reducing abilities that overcome the intense competition with other microorganisms for substrates and niches in the environment, undergone a drastic change in ORP and major substrates change under high alkaline conditions (pH 9.8–11.2). The importance of “PTS” was indicated in the later phase (maturation phase) in which the indigo fermentation consisted of two phases (Tu et al., 2021). Lopes et al. (2021) also reported the importance of “PTS” in fermentation, which accelerated indigo reduction by the addition of Indian indigo leaf powder during early fermentation. The importance of “starch and sucrose metabolism” in indigo reduction has been reported in comparative studies between of rapid and slow time-to-indigo reduction *sukumo* (Lopes et al., 2022). Using various preparation samples in this study, we clarified the significance of the two subpathways, “PTS” and “starch and sucrose metabolism,” by assessing the relationship between dyeing intensity and functional abundance during early fermentation. In addition, the earlier introduction of wheat bran brought about an earlier realization of high-intensity dyeing compared with the previous trials.

Two possible indigo reducing systems have been considered so far. The indigo particle reduction is induced by azoreductase (AzoA), which oxidizes NADH (Suzuki et al., 2018); however, this AzoA was found in *Alkalihalobacillus wakoensis* (similarity: 98.9%), which is not an essential member of the microbiota in *sukumo* fermentation. Therefore, there is a possibility that this reaction has negligible effect on indigo reduction during fermentation. Conversely, the acetate^−^/acetaldehyde couple is a strong electron donor than the NAD^+^/NADH couple (ORP = −0.601 V at pH10), with an ORP of −1.044 V at pH10. The acetate^−^/acetaldehyde coupled reaction can be catalyzed by (NAD-dependent) acetaldehyde dehydrogenases (Nakagawa et al., 2021). Acetaldehyde can be supplied from ethanol *via* the catalytic reaction facilitated by alcohol dehydrogenase. Another possible indigo reducing system is flavin-based extracellular electron transfer system found in *Listeria monocytogenes* (Light et al., 2018). The extracellular electron transfer is achieved *via* a series of intracellular electron transfers. NADH dehydrogenase (Ndh2) transfers electrons from NADH to the demethylmenaquinone (DMK) pool, which separates ordinary menaquinone (MK). Electrons are transferred from DMK to flavin adenine dinucleotide (FAD) on two FMNylased domain of PplA (outer membrane anchored electron transfer protein) or free flavin shuttles. Final electron acceptors, such as iron, accept electrons from FAD group on PplA or free flavin shuttles. This FAD-based extracellular electron transfer system expects widely distributed in Gram-positive bacteria. Seven NAD(P)-dependent oxidoreductases predicted using the PICRUSt2 analysis correlated with dyeing intensity were identified as the candidates for the initiation of indigo reductions in the KEGG orthologs level analysis. The intensity of these enzymes correlated with the initiation of indigo reduction in batches 3 and 4. These oxidoreductases, which are associated with the energy metabolism and the metabolisms of microbial metabolic products such as ethanol and lactic acid, were considered as candidate enzymes for reducing indigo particles.

Concerning the importance of favorable substrates, it has been reported that a period of accelerated convergence exists concurrent with a bloom of *Bifidobacterium* associated with the metabolism of oligosaccharides in breast milk (de Muink and Trosvik, 2018). The succession of microbiota and metabolic functional change in the microbial ecosystem influenced by environments and the substrates have been reported (Zhou et al., 2019; Kong et al., 2020). In addition to the pathways related to substrate metabolisms, the “prokaryotic defense system” was also identified as a pathway related to indigo reduction. The “prokaryotic defense system” is essential for defense against external DNA attachment by bacteriophages and exposure to antibiotics produced by the other bacterial in a highly competitive environment (Tajkarimi and Wexler, 2017; Alessandri et al., 2021). The abundance of bacteriophages in the gut microbiome has been reported (Carasso et al., 2020). These findings suggest that bacteriophages affect various microbial ecosystems. These findings and our analyses of the functional abundance ratios suggest that intense bacteria–bacteriophage or antibiotic relationships exist in the indigo fermentation system. The resulting microbiota that realizes indigo reducing state can survive a competition with other microorganisms for their nutrients and niches under the succession of the environmental changes.

Indigo fermentation is roughly divided into two phases (Tu et al., 2021). During the first phase, the microbiota changes relatively rapidly, while in the second phase, the changes stabilize. The first phase could be altered by the succession of decay and regeneration of the microbiota depending on their adaptabilities (e.g., high pH and low ORP) and the availabilities of substrates derived from *sukumo*. Our results of the functional prevalence for indigo reduction suggest that the substrate utilization and adaptation to the environment under the succession of the microbial community are important to access the indigo reduction state. The stable indigo reduced state is realized by the convergence of microbiota *via* a succession of decay and replacement of the microbial community from the initial community (first phase) and transformation into the second phase community in the presence of the limited substrates (mainly wheat bran) under alkaline environments. Besides the pretreatment of *sukumo*, continuous maintenance of high pH, low ORP, and introduction of wheat bran on day 5 during the maintenance of the fermentation fluid converged microbiota toward indigo reduction on day 7. This finding can be explained by RDA, the decrease in the ratio of “others,” which expresses miscellaneous low ratio constituent microorganisms, and the fluctuation in the alpha diversity change. This state may be the turning point from phase 1 (microbial community constructed after pretreatment of *sukumo*) toward phase 2 (relatively slow changing of the microbiota) (Tu et al., 2021). Previous studies have introduced wheat bran after day 22, which slowed the transition from phase 1 to phase 2 (Tu et al., 2019a,b, 2021). After day 22 of fermentation, the nutrition derived from *sukumo* was exhausted and the *sukumo* shifted to the phase of using nutrition from wheat bran. In other words, after fermentation begins and anaerobic microorganisms become dominant around day 3, the reduction of indigo starts around day 5, and after day 20, an ecosystem based on wheat bran is formed. This time, by introducing wheat bran during a highly flexible state of microbiota at the beginning of fermentation, the above stages were compressed, and even with different microbiota from different *sukumo* treatment, the effect of wheat bran started at day 6. A relatively strong indigo reduction occurs on day 6. This desirable state for indigo reduction was maintained until day 209. The long-term maintained state in the microbiota brought high-intensity of dyeing and a stable microbial community.

Indigo fermentation fluids exhibit exquisite resilience and maintain their indigo reducing ability for more than 1 year, even the fluids maintain under open air where there are many chances for contamination occur through immersed textiles (Okamoto et al., 2017; Aino et al., 2018). The first reason for the resilient ecosystem is the primary substrate in the fermentation which is wheat bran in this case. Wheat bran contains hard-to-utilizable substrates for microorganisms, such as starch, xylan, and cellulose. These hard-to-utilizable substrates produce slow changes and maintain a stable microbial community. If some bacteria contaminate the fermentation fluid, it is difficult for them to propagate using the hard-to-utilizable substrates under high pH and low ORP conditions. The second reason for the resilient ecosystem might be related to the microbiota formation process, which is converged by the succession of bacterial cells death and the regeneration of other taxa in the microbial community in the first phase. We suspect that the converged microbiota are maintained by the circulation of dead bacterial cells and the resulting generation of cell components that are utilizable microorganisms, which will lead the converged microbiota to adapt to the anaerobic alkaline environment. Some of the important functions of resistance to transitional changes in microbiota are shared in the converged microbial community. In addition, the microbial community exists in an anaerobic alkaline environment in the presence of limited substrates that exclude ordinary microorganisms. Therefore, the microbial community can converge easier than ordinary microbial community. The third reason for the stability of this ecosystem is that the stable ecosystem is based on the sharing of indispensable functions for indigo reduction among the major constituent microorganisms and obligate and facultative anaerobic acid producing bacteria. It is thought that the facultative anaerobic acid producing bacteria (e.g., *Amphibacillus* and *Alkalibacterium*) highly contribute to indigo reduction. In addition, these taxa are necessary to maintain this ecosystem. The predicted functional abundance suggests that obligate anaerobes utilize dead cells, which are produced by the pretreatment of *sukumo* and the initial transitional changes in microbiota owing to the high pH and the drastic decrease in ORP. This is in accordance with previous reports that described these obligate anaerobes-related taxa observed in this system such as *Alcalicella* (formerly *Anaerobrancaceae* or *Proteinivoraceae*) and *Tissierella* exhibited the ability for sludge degradations or bacterial cell lysis (Maspolim et al., 2015; Boltyanskaya and Kevbrin, 2016; Huang et al., 2018; Lavrentyeva et al., 2019; Wang et al., 2019). Dead cells are produced from the bacterial cells that died in the microbiota during the long-term fermentation period. Lactic acid bacteria utilize carbohydrates originating from wheat bran. Thus, a circulated system was constructed among the microbiota involved. The fourth reason is the functional redundancy among the constituted taxa. Several functions are necessary to maintain the circulation of this ecosystem, which exhibits simultaneously produces extracellular electrons *via* carbohydrate metabolisms. In this study, we identified functional redundancy between the same categories of the microorganisms such as obligate anaerobes and lactic acid bacteria. Resilient microbial systems have been found in other microbial communities due to their functional redundancy (Weimer, 2015; Luan et al., 2020; Zhao et al., 2021). The sharing of important functions for indigo reduction and the maintenance of this fermentation system were illustrated *via* the predicted functional abundance in major members of the microbiota. Furthermore, these important functions are shared in the predominant taxa.

Anaerobic fermentation systems with high pH can be observed in other ecosystems. Anaerobic alkaline conditions limit the survival of microorganisms; therefore, only limited members of alkaliphiles that can adapt anaerobic environment can be present. However, the core microorganisms change based on the substrates in the conditions. Proteins are major substrates; indeed, proteins such as those in fermented fish, *Tissierella* stains are core microorganisms (Osimani et al., 2019). Meanwhile, in plant derived materials treated with salt, such as green table-olive fermentation, *Alkalibacterium*, *Halolactibacillus*, and *Marinilactibacullus* are major members in the microbiota (Lucena-Padrós and Ruiz-Barba, 2016). A cementitious geological disposal facility for radioactive wastes provides a niche for microorganisms that can survive in hyperalkaline conditions. In the environment, formation of flocs composed of a complex mixture of extracellular polymers has been reported. *Alishewanella* and *Dietzia* were dominated in the flock (Charles et al., 2017). Some of the taxa mentioned above have also been found in indigo fermentation liquids. Compared with neutralophilic environments, possible members in the alkaline environments are limited. The members of ecosystems in alkaline environments may therefore be predictable according to their physicochemical conditions and nutrient availability for microorganisms. In *sukumo* fermentation, most microorganisms are derived from *sukumo*, which is itself derived from plant. However, it is produced solid fermentation on the in-house earthen floor. Gram-positive microorganisms are predominant in earthen floor, which may explain the predominance of Gram-positive bacteria in the indigo fermentation fluid. Gram-negative bacteria possess higher adaptability in aqueous alkaline environments than Gram-positive bacteria (Takebayashi et al., 2007). Therefore, there is a predominance of Gram-negative bacteria in the aged fermentation for indigo reduction (Lopes et al., 2021; Tu et al., 2021).

## Conclusion

*Sukumo* the composted leaves of *Polygonum tinctorium* L., provides autochthonous microorganisms and indigo dye to indigo reducing fermentation. The treatment procedure of *sukumo* is influenced by the artisanal practice. Although the aim of this treatment is to activate and select of microorganisms, the relationship between the involved environmental factors and microbiota remains unclear. *Sukumo* was treated in 4 different conditions and transitional change of the microbiota was analyzed. During days 2–5, before the effects of wheat bran were observed, treatment with high pH had higher impact than high temperature, while each pretreatment batch exhibited its own microbiota. Transitional changes in microbiota in alkaline treated *sukumo* were more rapid than those in non-alkaline-treated batches at the beginning of fermentation. High pH and low ORP altered the microbiota until day 5. Although the microbial community differed based on the treatment until day 5, each microbiota converged toward indigo reducing state at day 7 by the effect of the added wheat brane at day 5. *Amphibacillus*, *Alkalicella*, *Atopostipes*, and *Tissierellaceae* were core members at day 7. The relationship between the predicted functional abundance in the microbiota and the differences in the dyeing results between batches suggests that transportation and metabolisms of carbohydrates are important for initiation and acceleration of indigo reduction. Seven NAD(P)-dependent oxidoreductases with high correlation with indigo reduction intensity were identified as candidate enzymes for the extracellular electron transportation. The continuous addition of slowly decomposing wheat bran and the functional redundancy and successive changes of different indigo reducing taxa, which sustained the circulations of the ecosystem based on the fermentation aging, attributed to the sustainability of indigo reducing state. In this study, we demonstrated that alkaline treatment of *sukumo* and the addition of wheat bran are important for the secure realization of indigo reduction and maintenance at later stages of fermentation. In the future, adjustments for accurate alkaline pH and temperature for pretreatment of *sukumo* along with effective timing and more appropriate amount of wheat bran for early realization and maintenance of indigo reduction and to optimize conditions, better operation and performance of indigo fermentation can be accomplished.

## Data availability statement

The datasets presented in this study can be found in online repositories. The names of the repository/repositories and accession number(s) can be found at: https://www.ddbj.nig.ac.jp/, DRA014811.

## Author contributions

NF and IY: conceived and designed the experiments. NF, ZT, and HF: performed the experiments. IY, ZT, and HF: analyzed the data. IY: wrote the manuscript. All authors contributed to the article and approved the submitted version.

## Funding

This work was supported by the Institute for Fermentation (IFO), Osaka (G-2020-3-035; I.Y.).

## Conflict of interest

The authors declare that the research was conducted in the absence of any commercial or financial relationships that could be construed as a potential conflict of interest.

## Publisher’s note

All claims expressed in this article are solely those of the authors and do not necessarily represent those of their affiliated organizations, or those of the publisher, the editors and the reviewers. Any product that may be evaluated in this article, or claim that may be made by its manufacturer, is not guaranteed or endorsed by the publisher.

## Footnotes

^1^https://blast.ncbi.nlm.nih.gov/Blast.cgi

^2^https://huttenhower.sph.harvard.edu/galaxy/
