# Supplementary material for: Environmental factors contributing to the convergence of bacterial community structure during indigo reduction
Source: Front Microbiol. 2023 Feb 9;14:1097595. doi: 10.3389/fmicb.2023.1097595 (PMC9978934; doi:10.3389/fmicb.2023.1097595)
Supplement: Supplementary file 1 [file Data_Sheet_1.pdf]

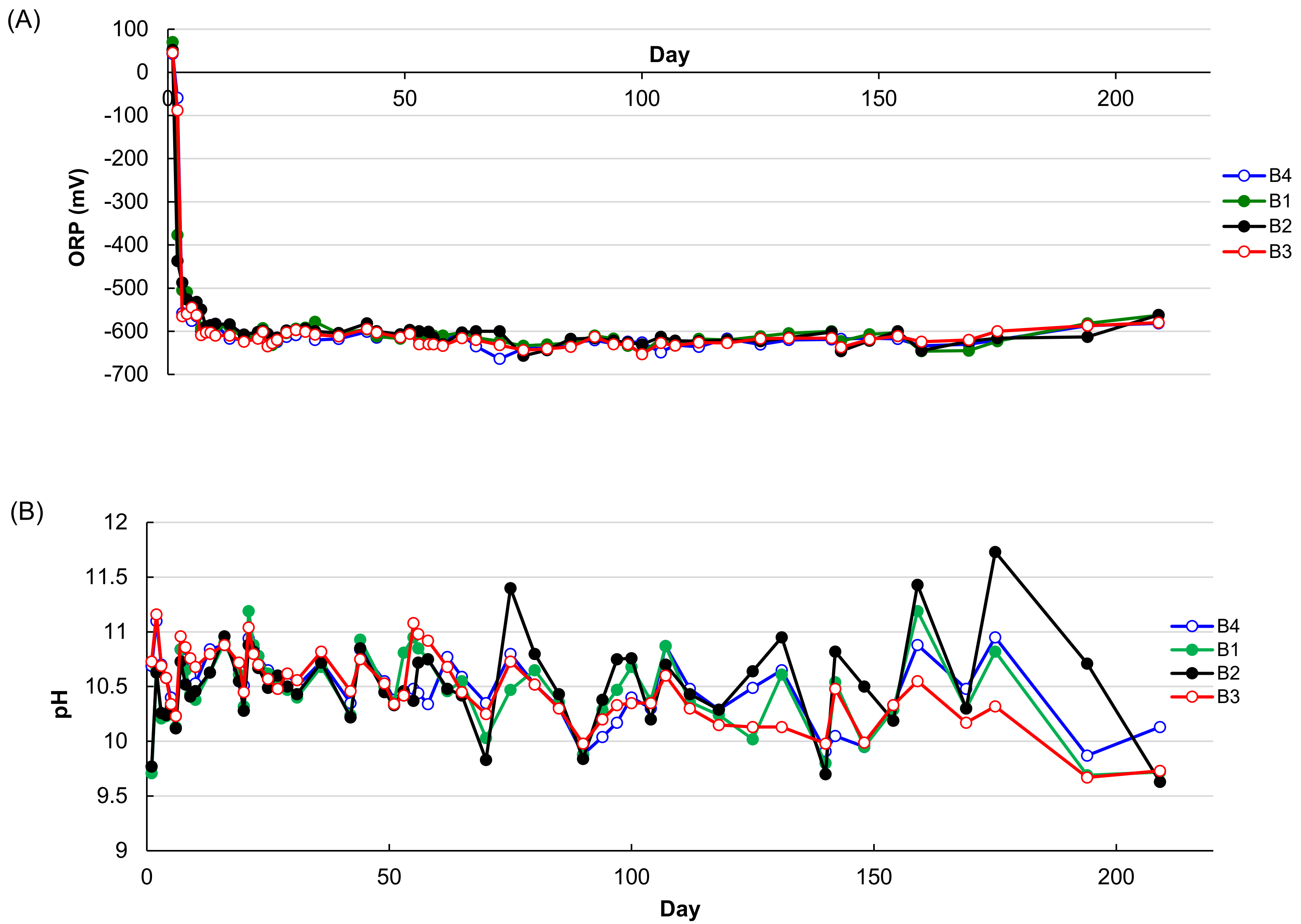

(A)
100
0
-100
-200
-300
-400
-500
-600
-700
ORP (mV)
Day
0
50
100
150
200
B4
B1
B2
B3
(B)
12
11.5
11
10.5
10
9.5
9
pH
0
50
100
150
200
Day
B4
B1
B2
B3

Supplementary Figure S1. Changes in redox potential (ORP) (A) and pH (B) depend on the fermentation period in indigo fermentation following different initial treatments of *sukumo*. B1: batch 1 (*sukumo* treated with 60°C tap water); B2: batch 2 (control; *sukumo* treated with 25°C tap water); B3: batch 3 (*sukumo* treated with 25°C wood ash extract); B4: batch 4 (*sukumo* treated with 60°C wood ash extract).

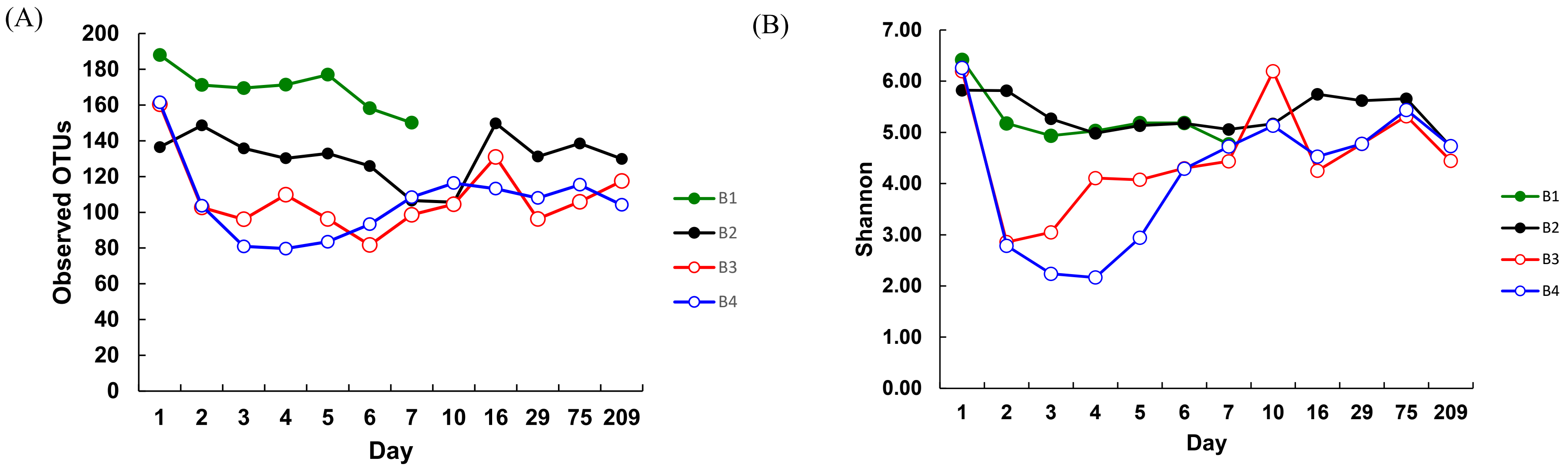

Supplementary Figure S2. Changes in alpha diversity (A): Observed operational taxonomic units (OTUs). (B) Shannon index of alpha diversity depending on the fermentation period analyzed using the Divisive Amplicon Denoising Algorithm (DADA2) based on the fermentation period. The 16S RNA sequencing depth was 8,825 for each sample. The number corresponding to the depth was calculated according to the read number for the coverage of all samples. The values are the average of 10 random sampling calculations. The standard deviations are less than 0.99 in the observed OTUs and less than 0.01 in Shannon index. Abbreviation: B, batch.

**Category**/Super Pathway	Sub Pathway	Day 3		
		B3/B1	B3/B2	B3/B4
**Cellular Processes**				
Cell motility:	Flagellar assembly	1.03	1.07	1.00
Transport and catabolism:	**Prokaryotic Defense System**	1.19	1.19	1.07
**Environmental Information Processing**				
Membrane transport:	**Phosphotransferase system (PTS)**	1.23	1.24	1.82
**Genetic information processing**				
Replication and repair:	DNA repair and recombination proteins	1.05	1.03	1.06
Translation:	Ribosome	1.07	1.04	1.07
	Translation factors	1.06	1.03	1.06
**Metabolism**				
Amino acid metabolism:	Phenylalanine, tyrosine and tryptophan biosynthesis	1.07	1.02	1.07
Carbohydrate metabolism:	Glycolysis / Gluconeogenesis	1.09	1.06	1.16
	**Starch and sucrose metabolism**	1.21	1.25	1.35
Metabolism of cofactors and vitamins:	Nicotinate and nicotinamide metabolism	1.12	1.11	1.08
**Unclassified**				
Genetic information processing:	Replication, recombination and repair proteins	1.09	1.05	1.11

Color scale: 2.00, 1.20, 1.05, 1.00

Supplementary Figure S3. The functional abundance ratio (B3/B1, B3/B2, and B3/B4) between the stained sample (B3) and the other unstained samples (B1, B2, and B4) on day 3. The metagenomic predictions produced using PICRUSt2 and BURRITO are shown. Subpathways containing a number > 1.06 in either B3/B1, B3/B2, or B3/B4 were selected. Abbreviation: B, batch.

Category/Super Pathway:	SubPathway	B2				B3				B4				
		D10/D2	D16/D2	D10/D5	D16/D5	D10/D2	D16/D2	D10/D3	D16/D3	D10/D2	D16/D2	D10/D4	D16/D4	
**Cellular Processes**														
Cell motility:	Flagellar assembly	1.41	1.31	1.24	1.15	1.26	1.26	1.23	1.23	1.28	1.25	1.14	1.11	
	Bacterial chemotaxis	1.41	1.26	1.18	1.06	1.21	1.18	1.18	1.16	1.20	1.19	1.04	1.04	
	Bacterial motility proteins	1.38	1.25	1.19	1.09	1.18	1.21	1.16	1.19	1.21	1.16	1.08	1.03	
Transport and catabolism:	**Prokaryotic Defense System**	1.42	1.38	1.37	1.32	1.25	1.21	1.23	1.20	1.30	1.32	1.18	1.21	
**Environmental Information Processing**														
Membrane transport:	**Phosphotransferase system (PTS)**	1.33	1.44	1.46	1.59	1.74	1.20	1.47	1.01	1.58	2.39	2.09	3.17	
**Genetic Information Processing**														
Replication and repair:	DNA replication proteins	1.13	1.11	1.11	1.09	1.13	1.11	1.10	1.09	1.13	1.14	1.11	1.12	
Translation:	Aminoacyl-tRNA biosynthesis	1.14	1.13	1.14	1.12	1.14	1.13	1.12	1.11	1.20	1.18	1.11	1.09	
	Ribosome	1.17	1.15	1.18	1.16	1.19	1.17	1.16	1.14	1.26	1.26	1.14	1.14	
	Ribosome biogenesis	1.15	1.12	1.13	1.10	1.17	1.15	1.15	1.13	1.19	1.20	1.13	1.13	
	Translation factors	1.16	1.14	1.15	1.13	1.16	1.14	1.14	1.12	1.25	1.24	1.11	1.10	
**Metabolism**														
Amino acid metabolism:	Lysine biosynthesis	1.15	1.13	1.12	1.10	1.10	1.14	1.09	1.14	1.20	1.14	1.07	1.01	
	Phenylalanine, tyrosine and tryptophan biosynthesis	1.05	1.01	1.11	1.08	1.18	1.14	1.17	1.12	1.22	1.25	1.14	1.16	
Carbohydrate metabolism:	Amino sugar and nucleotide sugar metabolism	1.02	1.01	1.05	1.04	1.26	1.10	1.23	1.07	1.16	1.32	1.21	1.39	
	Glycolysis / Gluconeogenesis	1.01	1.05	1.09	1.12	1.15	0.98	1.11	0.95	1.07	1.24	1.11	1.30	
	**Starch and sucrose metabolism**	1.23	1.25	1.29	1.31	1.38	1.05	1.25	0.95	1.21	1.56	1.36	1.76	
Metabolism of cofactors and vitamins:	Nicotinate and nicotinamide metabolism	1.09	1.05	1.14	1.10	1.14	1.12	1.12	1.10	1.20	1.20	1.14	1.15	
**Unclassified**														
Genetic information processing:	Replication, recombination and repair proteins	1.12	1.11	1.11	1.09	1.09	1.09	1.07	1.06	1.21	1.22	1.10	1.10	3.0
Metabolism:	Amino acid metabolism	1.22	1.19	0.98	0.95	0.61	0.69	0.63	0.71	0.71	0.60	0.51	0.44	2.0
	Carbohydrate metabolism	0.74	0.78	0.84	0.88	0.96	0.89	0.96	0.89	0.91	1.06	1.27	1.48	1.0
	Energy metabolism	1.26	1.20	1.16	1.10	1.02	1.00	1.02	1.01	1.10	1.08	0.86	0.84	0.5
	Others	0.77	0.84	0.91	0.99	1.04	0.93	1.04	0.92	0.86	1.00	1.15	1.34	

Supplementary Figure S4. The functional abundance ratio (D10/D2 and D16/D2) between stained samples (D10 and D16) and unstained sample (D2) in each batch (B2–3) and the ratio (D10 and day D16/D3–5) between stained samples and the samples' stain initiation (D3–5) in each batch. The metagenomic predictions produced using PICRUSt2 and BURRITO are shown. Subpathways containing a number > 1.13 in either ratio were selected. Abbreviations: B, batch; D, day. Blue font indicates the day dyeing initiated.

Category/SuperPathway	SubPathway	B1-D3 (%)			B2 (control)-D3 (%)				B3-D3 (%)				B4-D3 (%)		
		Function abundance	*Alkaliphilus* (41.6%)*	*Bacillus* (12.1%)	Function abundance	*Alkaliphilis* (46.2%)	*Bacillus* (6.8%)	*Paenibacillus* (4.2%)	Function abundance	*Alkaliphilus* (67.4%)	*Sutcliffiella* /*Bcillus* (7.9%)	*Alkalihalobacillus* (7.2%)	Function abundance	*Alkaliphilus* (80.5%)	*Bacillus* (9.5%)
**Cellular Processes**															
Cell motility:	Bacterial chemotaxis	0.67	11.5	33.6	0.63	16.0	22.7	16.8	0.69	29.1	22.5	23.5	0.71	43.4	39.6
	Bacterial motility proteins	1.90	10.5	33.8	1.80	14.6	23.1	14.4	1.95	26.7	26.3	22.6	2.02	39.7	42.2
	Flagellar assembly	0.74	9.8	32.8	0.71	13.3	22.7	14.5	0.76	24.9	26.1	22.8	0.76	38.1	42.4
Transport and catabolism:	**Prokaryotic Defense System**	**0.68**	10.8	31.0	**0.68**	14.2	21.5	7.3	**0.80**	23.8	26.8	15.7	**0.75**	39.0	40.8
**Environmental Information Processing**															
Membrane transport:	**Phosphotransferase system (PTS)**	**0.26**	1.2	40.9	**0.26**	1.5	25.1	10.3	**0.32**	2.5	20.3	42.5	**0.18**	7.0	56.5
**Genetic Information Processing**															
Replication and repair:	DNA replication proteins	0.73	8.1	33.5	0.73	10.6	22.4	13.1	0.75	20.6	26.3	21.7	0.72	32.8	45.3
Translation:	Aminoacyl-tRNA biosynthesis	0.65	9.1	31.9	0.66	11.7	20.5	13.1	0.68	22.7	24.5	18.7	0.65	36.2	41.2
	Ribosome	3.70	9.2	30.5	3.82	11.6	19.9	11.9	3.98	22.3	23.0	20.3	3.72	36.4	40.2
	Ribosome biogenesis	2.93	8.3	31.2	2.97	10.7	20.5	14.8	3.02	21.1	25.9	20.4	2.91	33.5	44.0
	Transfer RNA biogenesis	2.63	9.2	32.2	2.65	11.9	21.2	13.3	2.71	23.3	25.5	18.8	2.66	36.4	41.7
	Translation factors	0.83	9.7	30.9	0.85	12.4	20.1	12.9	0.88	24.1	22.5	19.2	0.83	38.8	38.3
**Metabolism**															
Amino acid metabolism:	Lysine biosynthesis	0.62	10.4	31.8	0.62	13.6	20.9	11.2	0.63	26.8	22.1	18.1	0.63	41.2	36.8
	Phenylalanine, tyrosine and tryptophan biosynthesi	1.05	8.3	30.7	1.09	10.5	20.1	12.4	1.12	20.5	24.1	21.5	1.05	33.4	42.5
Carbohydrate metabolism:	Amino sugar and nucleotide sugar metabolism	0.96	6.1	31.1	0.99	7.8	19.9	18.9	0.96	16.0	26.2	20.6	0.86	27.7	46.0
	Glycolysis / Gluconeogenesis	0.52	6.9	34.7	0.53	8.8	21.6	12.4	0.56	16.6	25.3	22.1	0.49	29.5	45.1
	**Starch and sucrose metabolism**	**1.03**	5.1	38.4	**0.99**	6.9	24.4	13.9	**1.24**	11.0	24.6	33.4	**0.92**	23.0	52.4
Metabolism of cofactors and vitamins:	Nicotinate and nicotinamide metabolism	0.65	8.6	33.5	0.66	11.1	21.8	12.0	0.73	20.0	25.2	23.6	0.68	32.9	45.0
**Unclassified**															
Genetic information processing:	Replication, recombination and repair proteins	0.99	9.2	30.5	1.03	11.7	21.0	13.0	1.08	22.1	22.0	24.0	0.98	37.6	39.0
Metabolism:	Amino acid metabolism	0.54	27.3	28.4	0.55	34.6	18.4	6.1	0.69	55.3	17.6	6.5	0.86	67.8	22.0
	Carbohydrate metabolism	0.57	2.2	37.6	0.56	2.8	25.6	18.0	0.55	5.8	31.8	24.2	0.45	11.1	59.6
	Energy metabolism	1.25	15.6	30.8	1.26	20.3	20.0	8.8	1.38	37.0	19.1	14.0	1.44	54.3	28.0
	Others	0.83	2.9	41.8	0.84	3.8	27.3	11.3	0.81	7.9	35.8	18.3	0.76	13.1	61.3

60.0%
40.0%
20.0%
10.0%
5.0%
1.0%

Supplementary Figure S5. Functional abundance ratio (%) of the total subpathways and for the contributing ratio (%) of the major constituent taxa in the subpathways related to the initiation and enhancement of indigo reduction of day 3 (D3) samples in batches 1–4. *: the number in the parentheses is the existence ratio (%) for the microbiota. The metagenomic predictions produced by PICRUSt2 and BURRITO are shown. The important subpathways and the functional abundance ratios are indicated in bold letters.

Category/SuperPathway	SubPathway	B2 (control)-10 (%)							B3-D10 (%)					B4 -D10 (%)						
		Function abundance	*Alkalicella* (17.8%)*	*Umitella* (4.4%)	*Tisssierella* (3.7%)	*Alkaliphilus* (27.4%)	*Atopostipes* (8.6%)	*Amphibacillus* (11.3%)	Function abundance	*Alkalicella* (14.7%)	*Alkaliphilus* (23.7%)	*Aopostipes* (11.1%)	*Amphibacillus* (28.3%)	Function abundance	*Alkalicella* (20.6%)	*Tissierella* (9.3%)	*Alkaliphilus* (14.1%)	*Atopostipes* (4.1%)	*Amphibacillus* (21.1%)	
**Cellular Processes**																				
Cell motility:	Bacterial chemotaxis	0.78	17.1	0.4	14.6	7.1	9.9	24.9	0.82	12.4	5.4	10.6	51.9	0.80	16.9	13.5	3.2	4.2	28.5	
	Bacterial motility proteins	2.24	19.1	0.4	15.3	6.4	8.5	23.1	2.27	14.4	5.0	9.5	49.7	2.35	18.5	13.8	2.8	3.5	26.3	
	Flagellar assembly	0.91	19.2	0.4	13.3	5.7	12.6	22.0	0.93	14.3	4.4	13.9	46.9	0.95	18.9	12.2	2.5	5.3	25.6	
Transport and catabolism:	**Prokaryotic Defense System**	**0.99**	17.4	0.5	17.9	5.3	13.6	20.4	**0.99**	13.3	4.2	15.4	44.6	**0.96**	18.3	17.6	2.5	6.1	24.0	
**Environmental Information Processing**																				
Membrane transport:	**Phosphotransferase system (PTS)**	**0.36**	0.0	0.0	1.5	0.6	38.4	31.4	**0.48**	0.0	0.4	32.5	51.4	**0.31**	0.0	1.7	0.3	19.4	44.2	
**Genetic Information Processing**																				
Replication and repair:	DNA replication proteins	0.82	15.0	0.4	13.9	5.2	12.8	21.6	0.83	11.2	4.1	14.2	46.4	0.81	15.3	13.2	2.4	5.6	26.1	
Translation:	Aminoacyl-tRNA biosynthesis	0.76	18.6	0.4	13.2	5.5	13.0	19.3	0.76	14.3	4.5	14.8	42.5	0.77	18.9	12.5	2.5	5.6	23.2	
	Ribosome	4.56	18.7	0.3	12.3	5.3	13.6	20.2	4.63	14.0	4.2	15.1	43.4	4.60	19.0	11.6	2.4	5.9	24.2	
	Ribosome biogenesis	3.40	17.7	0.3	12.1	5.1	12.8	21.2	3.47	13.2	4.0	14.1	45.4	3.44	17.9	11.4	2.3	5.5	25.5	
	Transfer RNA biogenesis	2.99	18.4	0.4	12.7	5.8	12.3	19.5	2.97	14.1	4.7	14.0	42.9	3.02	18.6	12.0	2.7	5.3	23.5	
	Translation factors	1.00	18.7	0.4	13.0	5.8	13.1	19.5	1.00	14.3	4.6	14.9	42.7	1.00	19.1	12.4	2.7	5.7	23.5	
**Metabolism**																				
Amino acid metabolism:	Lysine biosynthesis	0.72	15.0	0.5	18.1	6.4	11.2	19.6	0.69	11.9	5.4	13.1	44.6	0.72	15.3	17.1	3.0	4.8	23.9	
	Phenylalanine, tyrosine and tryptophan biosynthesis	1.19	20.2	0.2	7.9	5.3	9.9	24.9	1.31	14.1	3.8	10.2	49.5	1.27	19.4	7.1	2.3	4.0	28.3	
Carbohydrate metabolism:	Amino sugar and nucleotide sugar metabolism	1.04	13.3	0.2	7.1	4.1	15.3	26.7	1.18	8.9	2.9	15.3	51.5	1.01	14.1	7.0	1.9	6.9	32.1	
	Glycolysis / Gluconeogenesis	0.57	12.2	0.2	7.5	4.5	19.3	23.2	0.63	8.4	3.3	19.7	45.7	0.53	13.4	7.7	2.2	9.0	29.1	
	**Starch and sucrose metabolism**	**1.24**	7.4	0.1	4.9	3.0	27.9	29.1	**1.55**	4.4	1.9	25.1	50.7	**1.15**	8.1	5.0	1.5	13.0	37.1	
Metabolism of cofactors and vitamins:	Nicotinate and nicotinamide metabolism	0.75	18.5	0.3	10.8	5.4	9.1	25.7	0.82	12.8	3.9	9.4	51.1	0.81	17.5	9.4	2.3	3.7	28.8	
**Unclassified**																				60.0%
Genetic information processing:	Replication, recombination and repair proteins	1.16	17.0	0.4	14.3	5.6	11.0	19.9	1.15	13.2	4.5	12.5	44.0	1.15	17.6	13.8	2.6	4.8	24.7	40.0%
Metabolism:	Amino acid metabolism	0.58	9.4	0.8	27.5	17.9	9.9	8.8	0.43	9.7	19.3	15.0	25.9	0.51	10.9	30.0	9.5	4.9	12.2	20.0%
	Carbohydrate metabolism	0.46	7.4	0.3	9.3	1.9	7.6	29.9	0.53	5.1	1.3	7.4	56.5	0.46	7.5	8.9	0.9	3.3	34.0	10.0%
	Energy metabolism	1.53	18.2	0.5	18.2	9.1	10.6	16.8	1.40	15.0	8.0	13.0	40.1	1.42	19.8	18.6	4.5	4.9	21.3	5.0%
	Others	0.70	6.1	0.1	4.6	2.5	16.5	29.3	0.84	3.8	1.7	15.6	53.6	0.71	6.2	4.3	1.1	7.1	35.1	1.0%

Supplementary Figure S6. Functional abundance ratio (%) of the total subpathways and for the contributing ratio (%) of the major constituent taxa in the subpathways related to the initiation and enhancement of indigo reduction at day 10 (D10) samples in batches 2–4 (B2–4B). *: the number in the parentheses is the existence ratio (%) for the microbiota. The metagenomic predictions produced by PICRUSt2 and BURRITO are shown. The important subpathways and the functional abundance ratios are indicated in bold letters.
